# A comprehensive analysis of pneumococcal two-component system regulatory networks

**DOI:** 10.1093/nargab/lqae039

**Published:** 2024-04-22

**Authors:** Jens Sivkær Pettersen, Flemming Damgaard Nielsen, Patrick Rosendahl Andreassen, Jakob Møller-Jensen, Mikkel Girke Jørgensen

**Affiliations:** Department of Biochemistry and Molecular Biology, University of Southern Denmark, Odense, Denmark; Department of Biochemistry and Molecular Biology, University of Southern Denmark, Odense, Denmark; Department of Clinical Microbiology, Odense University Hospital, Odense, Denmark; Department of Microbiology, ETH Zürich, Zürich, Switzerland; Department of Biochemistry and Molecular Biology, University of Southern Denmark, Odense, Denmark; Department of Biochemistry and Molecular Biology, University of Southern Denmark, Odense, Denmark

## Abstract

Two-component systems are key signal-transduction systems that enable bacteria to respond to a wide variety of environmental stimuli. The human pathogen, *Streptococcus pneumoniae* (pneumococcus) encodes 13 two-component systems and a single orphan response regulator, most of which are significant for pneumococcal pathogenicity. Mapping the regulatory networks governed by these systems is key to understand pneumococcal host adaptation. Here we employ a novel bioinformatic approach to predict the regulons of each two-component system based on publicly available whole-genome sequencing data. By employing pangenome-wide association studies (panGWAS) to predict genotype-genotype associations for each two-component system, we predicted regulon genes of 11 of the pneumococcal two-component systems. Through validation via next-generation RNA-sequencing on response regulator overexpression mutants, several top candidate genes predicted by the panGWAS analysis were confirmed as regulon genes. The present study presents novel details on multiple pneumococcal two-component systems, including an expansion of regulons, identification of candidate response regulator binding motifs, and identification of candidate response regulator-regulated small non-coding RNAs. We also demonstrate a use for panGWAS as a complementary tool in target gene identification via identification of genotype-to-genotype links. Expanding our knowledge on two-component systems in pathogens is crucial to understanding how these bacteria sense and respond to their host environment, which could prove useful in future drug development.

## Introduction

*Streptococcus pneumoniae* is a major human pathogen that may cause severe infections such as pneumonia, sepsis and meningitis. It colonizes several different human niches, including the upper respiratory tract, middle-ear, lung, bloodstream and meninges (1). Consequently, sensing of these different environments is critical to its niche adaptation and survival. Two-component regulatory systems (TCSs) are key bacterial signal transduction systems that play an important role in environmental adaptation. They generally consist of two proteins: a membrane-embedded histidine kinase (HK) and a cytoplasmic response regulator (RR). In the prototypical model of TCS activation, the HK senses an environmental stimulus and becomes activated via auto-phosphorylation of a conserved histidine residue (2). HKs respond to a variety of environmental stimuli, including temperature, pH, nutrient availability, oxygen levels, cell-envelope stress, and quorum sensing molecules (3,4). Upon activation, the HK transfers the phosphoryl group to a conserved aspartate residue on the receiver domain of the cognate RR, which leads to a conformational change that enables the RR to regulate target gene expression. While the prototypical model depicts a very linear signal transduction pathway, the regulatory networks of TCSs are often more complex and include cross-regulation at multiple levels (2,5). For example, some RRs regulate expression of a secondary TCS at the transcriptional level (6,7), and some HKs activate auxiliary proteins that modulate the activity of a secondary HK or a non-cognate RR (8–10). Additionally, activation of some RRs occur through HK-independent pathways by serine/threonine protein kinases or via redox sensing (11–13). TCS auto-regulation is also common (14), and may result in positive or negative feedback loops, either directly through transcriptional induction or repression of the TCS-encoded operon by the RR (15,16), or indirectly through post-transcriptional feedback regulation mediated by TCS-activated small non-coding RNAs (sRNAs) (17).

Due to their significant impact on bacterial growth, fitness, and pathogenicity, TCSs have been proposed as potential antibacterial drug targets (3,18). Thus, understanding the TCS regulatory networks and the signals activating them is crucial for future drug development. The significance of TCSs for bacterial pathogenicity is highlighted in the pneumococcus, where at least 8 TCSs are critical for pneumococcal pathogenesis (19). The pneumococcal genome encodes 13 TCSs in total, and a single orphan response regulator (RitR), the majority of which have been broadly characterized. Previously reported functions of all pneumococcal TCSs and identified target genes are summarized in Table 1.

**Table 1.** Overview of pneumococcal two-component systems

Two-component system	D39V locus_tag	Name^a^	Reported functions	Target genes
TCS01	*spv_1445–6*	(BceRS)	Antimicrobial peptide resistance^b^	*bceAB*
TCS02	*spv _1084–5*	VicRK	Cell envelope integrity and virulence^c^	*pcsB*, *pspA*, *spv_0104*, *spv_0703*, *spv_1874*
TCS03	*spv _0351–2*	VraSR	Cell envelope stress and self-lysis protection^d^	*vraTSR-alkD, spv_0355-cbpG-cbpK*, *spv_0803, spxA2*
TCS04	*spv _1908–9*	PnpRS	Regulation of inorganic phosphate homeostasis (Pho regulon)^e^	*pst1* operon
TCS05	*spv _0701–2*	CiaRH	Virulence, cell wall stress, and carbohydrate utilization^f^	*axe1, dltXABCD, htrA-parB*, *ccnA-E*, *malQP*, *manLMN*, *srf-21*, *tarIJ-licABC*
TCS06	*spv _2019–20*	CbpRS	Virulence^g^	*cbpA*, *pspA*
TCS07	*spv _0157–8*	(YesMN)	Host glycan metabolism^h^	See Andreassen *et al.*, 2020.
TCS08	*spv _0081–2*	(SaeRS)	Virulence, metabolic adaptation^i^	See Gómez-Mejia*et al.*, 2018.
TCS09	*spv _0574–5*	YesNM	Carbohydrate metabolism, extracellular oxidative stress^j^	See Hirschmann *et al.*, 2021.
TCS10	*spv _0524–5*	VncRS	Vancomycin tolerance^k^	*vex123*
TCS11	*spv _1798–9*	(DesKR)	Sugar utilization^l^	See Wang *et al.*, 2020
TCS12	*spv _2063–4*	ComDE	Competence, stress respone and virulence^m^	See Slager *et al.*, 2019.
TCS13	*spv _0468–9*	BlpRH	Bacteriocin production and immunity^n^	See de Saizieu *et al.*, 2000.
**Orphan response regulator**				
RR14	*spv_0344*	RitR	Iron uptake, redox sensing^o^	*piuBCDA*

^a^Names as annotated in D39V (except for TCS06 - here named CbpRS). TCSs with no annotated names are indicated by the names of homologous TCS from other organisms in parenthesis. ^b^(66). ^c^(27). ^d^(55). ^e^(67). ^f^(74,76). ^g^(68). ^h^(47). ^i^(80). ^j^(34), and this study. ^k^(52,81). ^l^(53). ^m^(43,82). ^n^(29,83). ^o^(12,54).

n.a.: information not available.

A total of 13 TCSs, and a single orphan response regulator are encoded by the D39V genome. The table lists their name, reported functions and target gene examples (for less validated or large TCS regulons, a relevant study is referenced).

The role of TCSs in host adaptation and virulence is well recognized in other pathogenic streptococcal species as well. However, variations in host and niche preferences among streptococci indicate their dependence on distinct two-component system (TCS) repertoires for detecting and adjusting to their specific surroundings. Still, several pneumococcal TCSs are conserved across multiple streptococcal species, including VicRK, CiaRH, BlpRH and ComDE (20–22). For instance, CiaRH is present in multiple human and animal pathogenic streptococci (including *S. pyogenes*, *S. agalactiae*, *S. gordonii, S. iniae* and *S. suis*) where it is involved with functions related to biofilm formation, virulence, and cell wall integrity (21). While differences in the target regulons of orthologous TCSs are typically observed, some target genes often recur across species. For CiaR, this includes the CiaR-dependent sRNAs (csRNAs), and the *dlt* operon involved with teichoic acid biosynthesis (23–25). Other examples include VicR-mediated regulation of the murein hydrolase PcsB in *S. pyogenes* and *S. pneumoniae* (26,27), BlpR-mediated regulation of genes involved with bacteriocin production and immunity in *S. mutans* and *S. pneumoniae* (28,29), and a large overlap in competence regulon genes regulated by ComE in the mitis and anginosus groups of streptococci (30–32). Understanding differences and similarities in functions and target regulons of orthologous TCSs of closely related streptococcal species could provide new insights into the role of the TCSs in pneumococcal pathogenesis.

It is evident that several pneumococcal TCS regulons are well-defined (e.g. CiaRH, BlpRH, and ComDE) while others remain vaguely characterized (e.g. TCS08, YesNM and TCS11). Moreover, much of our current knowledge on several TCS regulatory networks is based on older studies using microarrays, which fail to cover the whole transcriptome including many sRNAs. Furthermore, multiple studies have primarily investigated deletion mutants of either the HK, RR, or both (33–35), which potentially fails to detect regulatory effects under non-induced conditions. In this study, we perform a comprehensive analysis of the regulatory network of 12 pneumococcal TCSs, focusing on those least characterized. To do this, we use a pangenome-wide association study (panGWAS) approach to identify TCS co-occurring genes across streptococcal species, in combination with RNA-sequencing of RR overexpression mutants. Our analysis identifies instances of cross-regulation and self-regulation within two-component systems (TCS), uncovering both previously documented and newly discovered constituents within various pneumococcal TCS regulatory networks. We also demonstrate that the panGWAS approach is a strong complementary tool for TCS target gene identification via identification of genotype-genotype links.

## Materials and methods

### Bacterial growth conditions and construction of mutants

*Streptococcus pneumoniae* D39V and its derivatives were cultivated in semi-defined C + Y medium or on Columbia agar plates supplemented with 2% (v/v) defibrillated horse-blood (SSI-Denmark) at 37°C with 5% CO_2_. Creation of overexpression mutants was performed by homologous recombination using the chromosomal expression platform (CEP) for chromosomal insertion, described in Sorg *et al.* (36). This expression platform is integrated between *treR* and *amiF* on the pneumococcal chromosome and contains a strong constitutive promoter, here used for overexpression of the response regulators. Through PCR, an upstream DNA fragment containing a ∼0.5 kb homologous region, spectinomycin resistance gene and the promoter region was amplified (using primer #1 + 2), together with a downstream ∼ 0.6 kb homologous region (using primer #3 + 4). In parallel, the response regulator sequences were amplified using primers with overhangs corresponding to the reverse complementary sequence of the reverse and forward primers used for amplifying the upstream and downstream regions, respectively (primers #5–28, primers listed in Supplementary Table S1). Subsequently, overlap PCR was then used to fuse the PCR fragments together creating a single linear PCR fragment. Transformation was performed by inducing pre-competent pneumococcal cells with 0.2 μg/ml competence stimulating peptide (CSP) for 12 min at 37°C, followed by incubation with the linear PCR products for 20 min at 30°C. Finally, transformants were incubated at 37°C for at least 1.5 h before spreading on Columbia blood agar plates and incubated overnight (ON). For selection of overexpression mutants, 100 μg/ml spectinomycin was used. The full list of mutants constructed for this study is available in the supplementary material (Supplementary Table S2). All strains were confirmed by PCR (using primer #56 + 67), and the resulting PCR fragments were sent for sequencing at Eurofins using Mix2Seq Kits.

### RNA extraction

Extraction of RNA from pneumococcal cell pellets was carried out by phenol–chloroform extraction. Briefly, total RNA for RNA-sequencing was extracted from cultures terminated in liquid nitrogen. Cell pellets were resuspended in cold solution 1 (10 mM Na-Citrate, 10 mM Na-acetate pH 4.5. and 2 mM EDTA) and transferred to a mixture of 150 μl solution 2 (10 mM Na-acetate pH 4.5, and 2% SDS), 700 μl acidic phenol (pH 4.5) and 300 μl chloroform. Tubes were then inverted and heated at 80°C for 4 minutes, followed by cooling on ice. Subsequently, tubes were centrifuged at 10 000×g for 5 min, and the aqueous phase was transferred to 96% ethanol with Na-acetate (37.5 mM) and precipitated ON. RNA was pelleted by centrifugation (20 000×g for 45 minutes), and washed in ice-cold ethanol. RNA pellets were resuspended in RNase-free H_2_O and stored at −20°C or −80°C.

### RNA-sequencing and data analysis

D39V wild-type and response regulator overexpression mutants (CEP::*rr01*, CEP::*rr03*, CEP::*rr04*, CEP::*rr05*, CEP::*rr06*, CEP::*rr08*, CEP::*rr09*, CEP::*rr10*, CEP::*rr11*, CEP::*rr13*, CEP::*rr14)* were grown in rich C + Y medium to an OD_600_ 0.4 in biological triplicates and harvested for RNA-sequencing. Ribosomal RNA was depleted from total RNA using the NEBNext rRNA Depletion Kit (Bacteria) (New England BioLabs). RNA-sequencing library preparation was carried out with NEBNext Ultra II Directional RNA Library Prep Kit for Illumina (New England Biolabs). Paired-end sequencing was performed on a NovaSeq 6000 System (Illumina). Raw paired-end reads were mapped to the *S. pneumoniae* D39V chromosome (GenBank: CP027540.1) with Bowtie2 using local alignment. Reads were counted inside features with *featureCounts* from the Subread package using a modified General Feature Format (gff) file for CP027540.1, which included annotations of sRNAs identified in D39W correctly fitted to corresponding positions in D39V. Reads were assigned to features with largest overlap, and multimapping reads were counted using a fractional count (1/*n*, where *n* is the total number of alignments reported). Differential expression analysis was performed in R using EdgeR. Genes with a |log_2_ (FC)| > 2 and FDR < 0.05 were defined as significantly differentially expressed. Clustered heatmaps were made with the R package *pheatmap*, using Ward's method (‘ward.D2’) for hierarchical clustering.

### Response regulator binding motif identification

DNA motif discovery was carried out with GLAM2 from the MEME Suite web tool (37) (version 5.5.2). Unless stated otherwise, at least 10 upstream sequences of 300 bp length from either different pneumococcal genes or homologous sequence regions from different streptococcal and non-streptococcal species were used for motif discovery. Identified conserved motifs showing a repeated sequence pattern (either direct repeat (DR) or inverted repeat (IR)) were used for proposed candidate motifs. The GLAM2-predicted candidate motifs were subsequently used as input to scan upstream sequences (400 bp) of RR-specific differentially expressed operons using FIMO from MEME Suite (filtered with a *P*-value < 0.001 using a default background model).

### Reverse-transcription quantitative PCR

Reverse-transcription quantitative PCR (RT-qPCR) was performed to validate response regulator overexpression in overexpression mutants (CEP::*rr01*, CEP::*rr03*, CEP::*rr04*, CEP::*rr05*, CEP::*rr06*, CEP::*rr08*, CEP::*rr09*, CEP::*rr10*, CEP::*rr11*, CEP::*rr13*, CEP::*rr14)* compared to wild-type grown in C + Y medium at 37°C to an OD_600_ 0.4, in biological triplicates. RNA was extracted as previously described. 1 μg RNA from each strain was treated with RNase-free DNase I (New England BioLabs) before cDNA synthesis was performed using the High-Capacity cDNA Reverse Transcription Kit (Applied Biosystems™, Thermo Fisher). Real time quantitative PCR was carried out with RealQ Plus 2x Master Mix Green (Ampliqon) using gene-specific primers on a LightCycler 480 System. Primers used are listed in Supplementary Table S1 (primers #29–55) and were designed to produce amplicons of 150–200 bp.

### TCS screening

The presence of each TCS was inferred by performing a tblastn for the HK/RR genes individually against each genome using blast+ v. 2.6.0 (38). The cut-off e-value was tested at increments of 10e-5 and its ability to distinguish the different TCSs was assessed, the final e-value cut-off was set to 10e-50 to return nearly identical proteins. A genome was considered to contain the specific TCS if a positive hit was detected within 1000 bp of each other regardless of order.

To ensure that no additional TCSs were encoded within the genome of *S. pneumoniae* D39V a screening was performed using the MiST3 database (39). The database consists of a non-redundant database of bacterial signal transduction sequence motifs and is used to detect genes encoding TCSs and other signal transduction systems.

### Pangenome-wide association study

Open reading frames of all genomes were predicted using Prokka v.1.13.3 at the standard parameters of the software (40). The annotated genomes were used to create a pangenome using Roary v. 3.12.0 and was set to create fast core gene alignment with MAFFT (-e), not to split paralogs into multiple gene clusters (-s), allow paralogs in the core genome alignment (-ap), percentage of isolates in which a gene had to be present to be a core gene to 90% (-cd) and cluster genes with a blastp sequence identity cut-of at 40% (-i) (41). The blastp sequence identity was identified by lowering the value at increments of 1% until known core genes were correctly clustered across the genus.

The pangenome was used as an input for Scoary v. 1.6.16 to perform a panGWAS analysis (42). Scoary uses the pangenome along with a trait matrix specifying which of the genomes within the pangenome contains a specific trait and scores all genes within the pangenome according to their co-occurrence with said trait. For this analysis the trait was set to be the possession of an intact pair of HK/RR genes and a trait matrix was created for each TCS. The presence of each HK/RR pair was identified as described previously. Finally, the ability of the panGWAS to correctly predict the co-occurrence of the TCS encoding genes with TCS itself confirmed that the low identity score set for the pangenome gene clustering was still in accordance with the high threshold set when screening for the TCSs as described previously.

## Results

### All pneumococcal two-component systems are highly conserved

**Figure 1.**
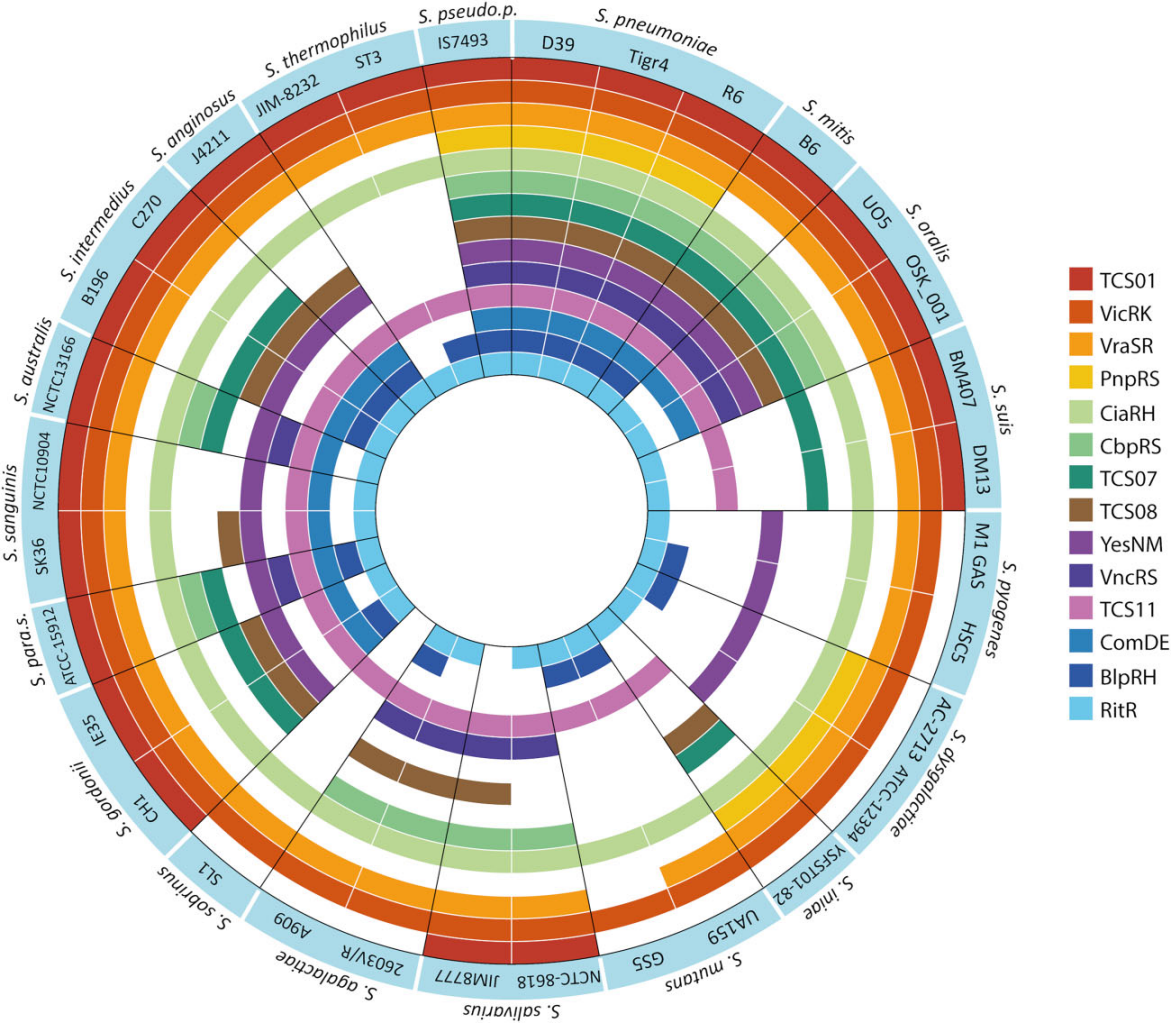
Distribution of the two-component systems (TCSs) of pneumococcus across selected *Streptococcus* strains. All TCSs are present in >99% of pneumococcus genomes and VicRK, VraSR, CiaRH and RitR are present in >99% of all *Streptococcus* genomes. The presence of each TCS is defined as a positive tblastn hit within 1000 bp of each other, of the translated aa sequence of each RR and HK genes from each TCS, the tblastn e-value cut-off was set at 10e-50.

We wanted to examine the individual pneumococcal TCSs and their distribution across pneumococcal isolates. A genomic screening revealed that all 13 TCSs and the orphan RR are present in >99% of pneumococcal genomes, making them part of the core genome. The screening was expanded to include the entire streptococcus clade, with 584 genomes across 33 unique species (Figure 1 and Supplementary Table S3). All pneumococcal TCSs were present in other streptococcal species, with VicRK, VraSR, CiaRH and RitR being present in >99% of streptococcus genomes, making them part of the core genome of the entire clade. Further screening determined that there were no undiscovered two-component systems (TCSs) within the *S. pneumoniae* D39V genome.

### Identification of TCS co-occurring genes using a panGWAS approach

**Figure 2.**
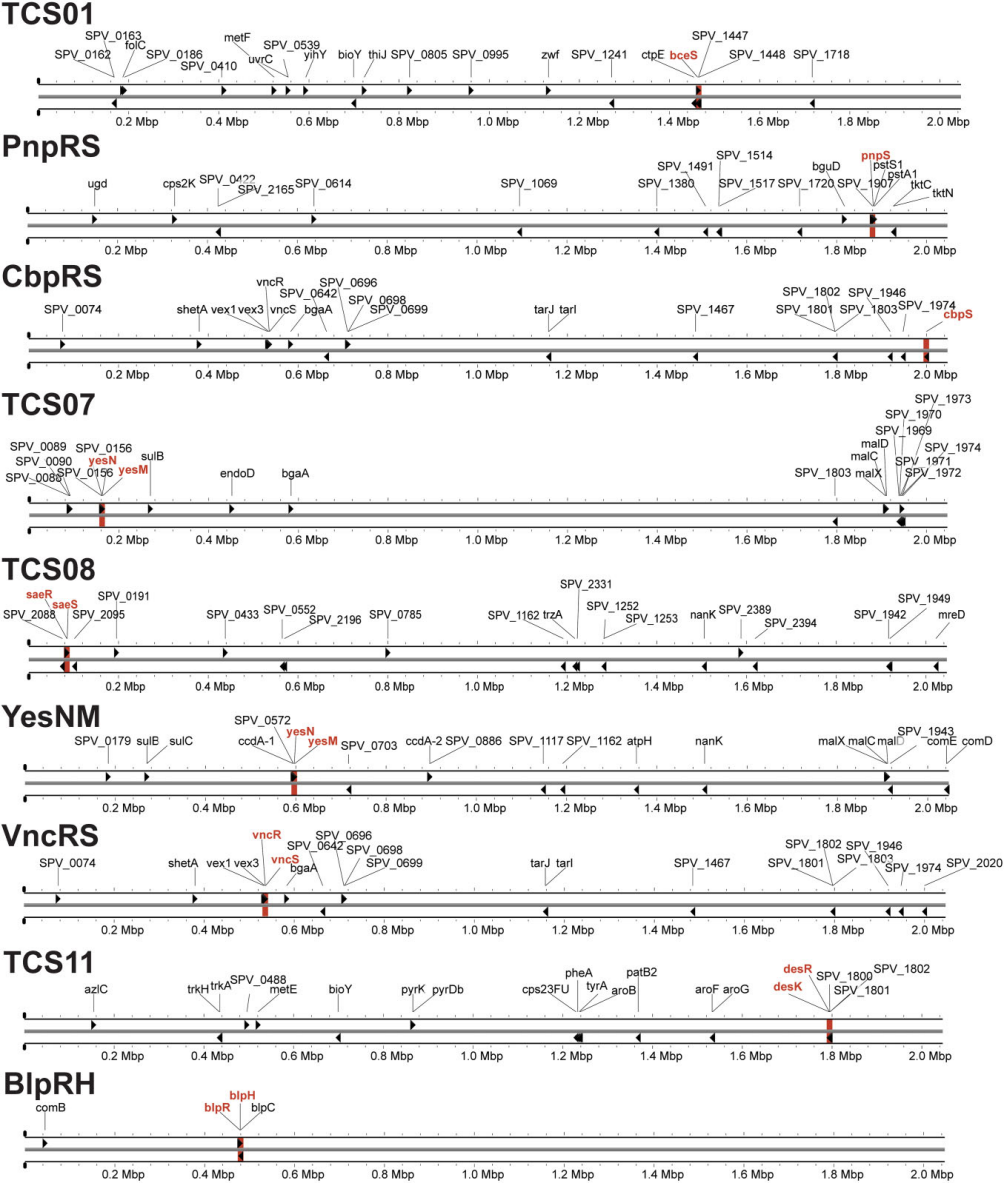
Genes co-occurring with each HK-RR pair across the streptococcal pangenome. Genes predicted to be associated with each TCS based on a pangenome-wide association study. Each gene is marked according to its location and annotation within the *S. pneumonia* D39V genome. A maximum of 20 hits are shown for each TCS. Genes encoding the HK/RR gene pairs themselves are shown in red if part of the prediction.

Originally, the panGWAS method was intended to link phenotypes to genotypes in bacteria (42). In this study, we applied the method to identify genotype-to-genotype links. Specifically, we utilized panGWAS to identify genes that significantly co-occur with each pneumococcal TCS across the entire streptococcal pangenome. As the panGWAS method requires genomes both with and without the analyzed trait, only the ten TCSs that are part of the streptococcus accessory genome could be analyzed (TCS01, PnpRS, CbpRS, TCS07, TCS08, YesNM, VncRS, TCS11, ComDE and BlpRH). We hypothesized that TCS target genes would co-occur with the genes encoding the specific RR and HK genes, thus providing an alternative method for TCS target identification. Since the study was focusing on the less characterized TCS of pneumococcus, ComDE was omitted from this study. A pangenome of all available *Streptococcus* assemblies (584 isolates distributed across 33 different species) was created resulting in a core genome of 839 genes and an accessory genome of 13 595 genes. The panGWAS analysis was then performed on each pneumococcal TCS to identify co-occurring genes (Figure 2). All genes predicted to co-occur with each TCS is listed in Supplementary Table S4.

### Transcriptomics corroborate the panGWAS TCS target identification approach.

**Figure 3.**
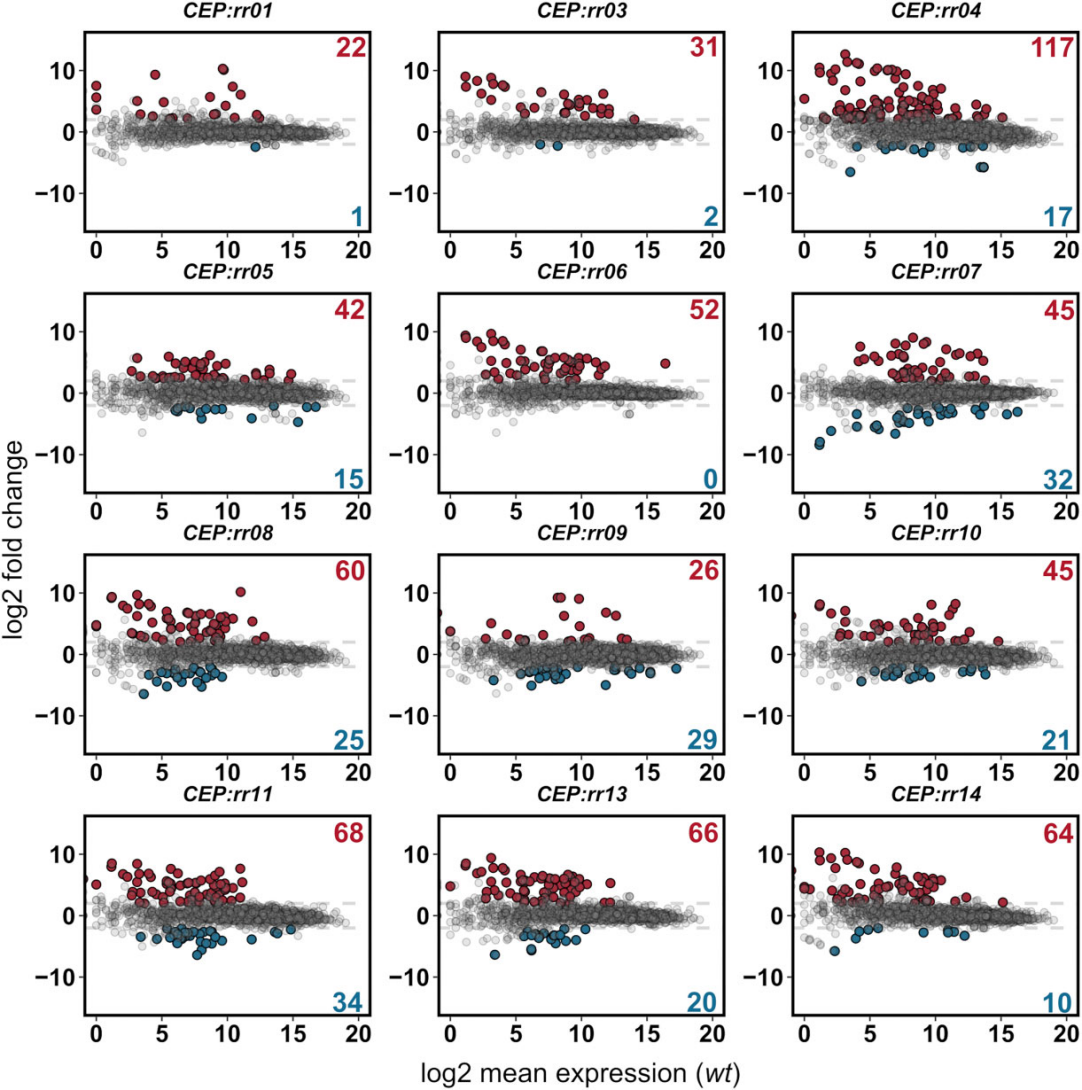
Transcriptomic response of *S. pneumoniae* to RR overexpression. MA-plots of differentially expressed genes in RR overexpression mutants. Significantly upregulated genes (log_2_ (FC) > 2, FDR < 0.05) are highlighted in red, and significantly downregulated genes (log_2_ (FC) ←2, FDR < 0.05) are highlighted in blue.

**Figure 4.**
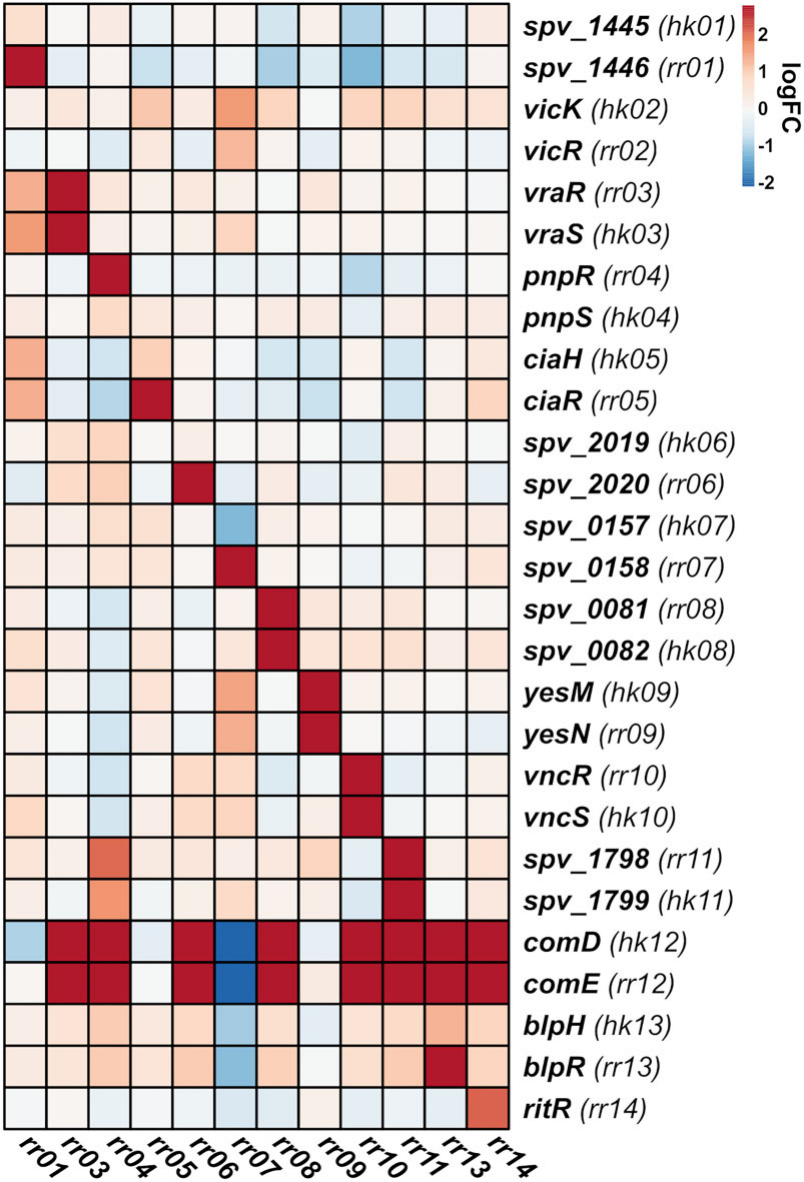
TCS cross-regulation in *S. pneumoniae*. Heatmap represent expression fold-change levels of pneumococcal RR and HK genes in CEP::*rr* overexpression mutants relative to the wild-type. Fold-change levels were derived from edgeR differential expression analysis of RNA-seq data. Heatmaps were constructed using the R-package ‘pheatmap’.

To validate TCS co-occurring genes as regulatory targets, transcriptomic analyses were conducted. For these analyses, chromosomal overexpression mutants were made in a *Streptococcus pneumoniae* D39V background, overexpressing 11 of the total 14 pneumococcal response regulator genes: *rr01*, *vraR*, *pnpR*, *ciaR*, *cbpR*, *rr08*, *yesN*, *vncR*, *rr11*, *blpR* and *ritR*. The VicRK and ComDE TCSs were omitted from this study. The ComDE TCS was not included as it has been studied extensively elsewhere (43–45). The VicRK TCS was omitted due to difficulties creating viable mutants overproducing the essential VicR response regulator. Since VicR is essential and involved with regulating cell wall metabolism and cell division (27,46), it is it possible that this is caused by VicR overproduction having detrimental effects on cell growth. The VicR regulon has previously been covered elsewhere (27). We recently covered TCS07 in a separate study but it was also included in the analyses of this study (47,48). Overexpression was carried out from the chromosomal expression platform (CEP) site using a strong constitutive promoter. All overexpression mutants were successfully confirmed by DNA sequencing, and reverse-transcription quantitative PCR (RT-qPCR) (Supplementary Figure S1). To investigate the transcriptome of each mutant, the overexpression mutants and the parental D39V strain were grown in rich C + Y medium to the mid-exponential growth phase (OD_600_ 0.4), and RNA was harvested and sequenced by Illumina RNA-sequencing (49). The total number of differentially expressed genes (|log2 (FC)| >2, FDR < 0.05) identified between the parental D39V strain and each of the 11 overexpression mutants, ranged from 23 in the *CEP::rr01* strain to 134 in the *CEP::rr04* strain (Figure 3, Supplementary Table S5). It was immediately noticed that multiple competence-induced genes were highly induced in several of the overexpression mutants. In fact, overexpression in 8 of the RRs resulted in induction of competence, as illustrated by the induction of the *comDE* operon (Figure 4). In pneumococcus, the competence system is not only involved with transformation but also acts as a general stress response. Thus, the observed competence induction may be an indirect effect caused by a stress response induced by RR-overexpression. The expression heatmap of pneumococcal TCS genes also revealed potential cross-regulatory events between other TCSs. For example, induction of *rr01* expression resulted in weak upregulation of the *vraRS* and *ciaRH* operons (log_2_ (FC) > 1, FDR < 0.05), and induction of *pnpR* expression resulted in weak upregulation of *TCS11* (log_2_ (FC) > 1, FDR < 0.05). Based on differential expression of cognate HK genes, it could also be determined which of the TCSs are auto-regulated. This included *vraRS*, *TCS08*, *yesMN*, *vncRS* and *TCS11*, which were all positively auto-regulated.

**Figure 5.**
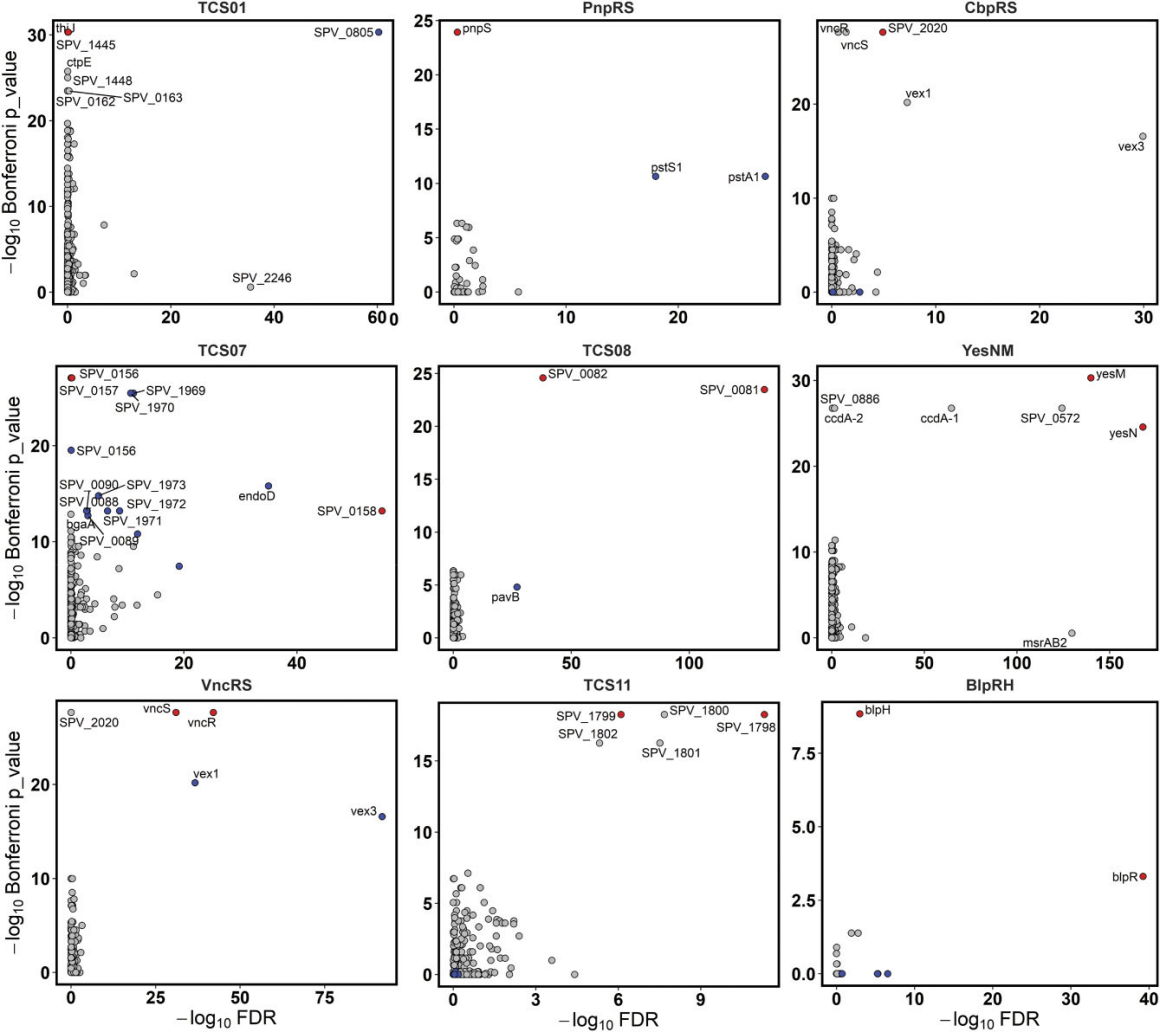
Significance of TCS-gene co-occurrence in relation to significance of differential expression in RR overexpression mutants. Genes co-occurring with each TCS across all streptococcal species as given by how significantly they co-occur (Bonferroni *P*-value) and their corresponding FDR value from the differential expression analysis in their respective RR overexpression mutants. Only TCSs that were found to be part of the *Streptococcus* clade accessory genomes could be analyzed with GWAS, which include TCS01, PnpRS, CbpRS, TCS07, TCS08, YesNM, VncRS, TCS11 and BlpRH. Previously identified target genes are highlighted in blue, and HK and RR genes are highlighted in red.

The identified co-occurring genes for each of the studied pneumococcal TCSs was compared directly to the transcriptomic data by comparing the level of significance for co-occurrence (Bonferroni *P*-values) to the level of significance for differential expression (FDR). As seen in Figure 5, in several cases the most significant co-occurring genes were also associated with significant differential expression in the respective RR overexpression mutant. Notably, the genes that display the highest level of differential expression in the overexpression mutants (e.g. *spv_0805* in CEP::*rr01*, *pst1S* and *pst1A* in CEP::*rr04*, and *ccdA1* and *etrx1*(*spv_0572*) in CEP::*rr09*) were also identified as the most significantly co-occurring genes in the panGWAS analysis (Supplementary Table S4 and Figure 5). In addition, the panGWAS analysis identified multiple genes with significant levels of co-occurrence, which were not differentially expressed in the RR overexpression mutants (e.g. *thiJ*, *ctpE*, and *spv_1448* for TCS01). Conversely, some differentially expressed genes showed non-significant levels of co-occurrence (e.g. *msrAB2* for YesNM). The former examples might represent genes that are associated with the TCS due to a functional, non-regulatory relationship, whereas the latter examples might represent genes specific to the TCS response in pneumococcus. In summary, these data demonstrate that the panGWAS approach identifies a strong co-occurrence between the TCS and their most strongly regulated targets, thus supporting findings from the transcriptomics-based TCS target identification approach, and demonstrate a novel bioinformatics approach to identify putative target genes of uncharacterized TCSs.

### An in-depth analysis of pneumococcal RR overexpression expands several TCS regulons

The transcriptomic analyses revealed a broad induction of competence- and stress response genes across several overexpression mutants. While this may represent cross-regulation with the ComDE system, it was difficult to distinguish which genes were specifically regulated by the respective RR. To address this challenge, we generated a clustered heatmap incorporating expression data from all overexpression mutants, allowing us to identify the genes specifically induced by each RR (example in Supplementary Figure S2), and to exclude genes being differentially expressed in multiple strains, as these might represent non-specific targets.

Using this clustering approach, it was possible to group differentially expressed genes in distinct clusters: RR-specific (i.e. genes with significant differential expression (|logFC| > 2, FDR < 0.05) in specific RR-overexpression mutants) and RR non-specific genes (i.e. genes with significant differential expression (|logFC| > 2, FDR < 0.05) in multiple RR-overexpression mutants). For RR01, this led to the identification of a previously identified TCS01 target, the *bceAB* operon, as a RR01-specific target. Other RR01-specific targets also included *pavB* (RR01- and RR08-specific) and an operon that is part of the *rtg* locus (Supplementary Figure S2) (50). According to the panGWAS, only the *bceAB* operon was identified as a candidate target, indicating that *pavB* and the *rtg* operon might be pneumococcus-specific RR01 targets.

**Figure 6.**
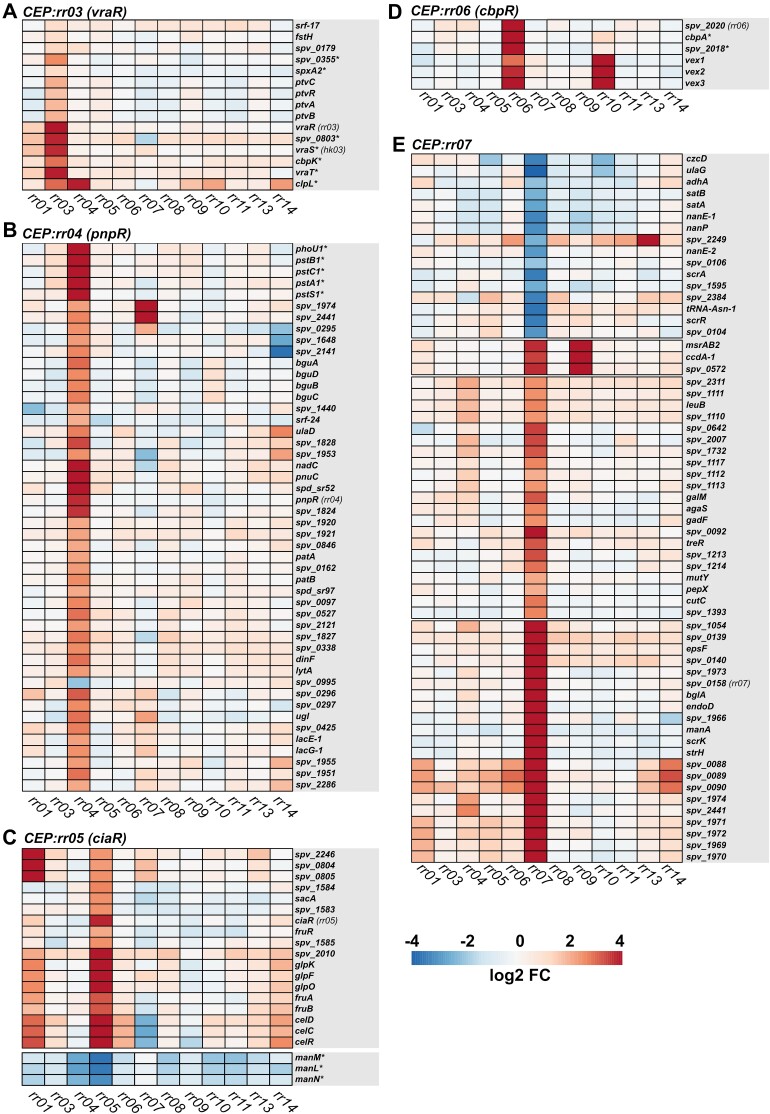
Identification of TCS regulons by RR overexpression – part 1. Clustered heatmaps of RR-specific differentially expressed genes (|logFC| > 2, FDR < 0.05) in RR overexpression mutants ((**A**) CEP::*rr03*, (**B**) CEP::*rr04*, (**C**) CEP::*rr05*, (**D**) CEP::*rr06*, (**E**) CEP::*rr07*). Previously identified TCS regulon genes are highlighted by an asterisk (*).

**Figure 7.**
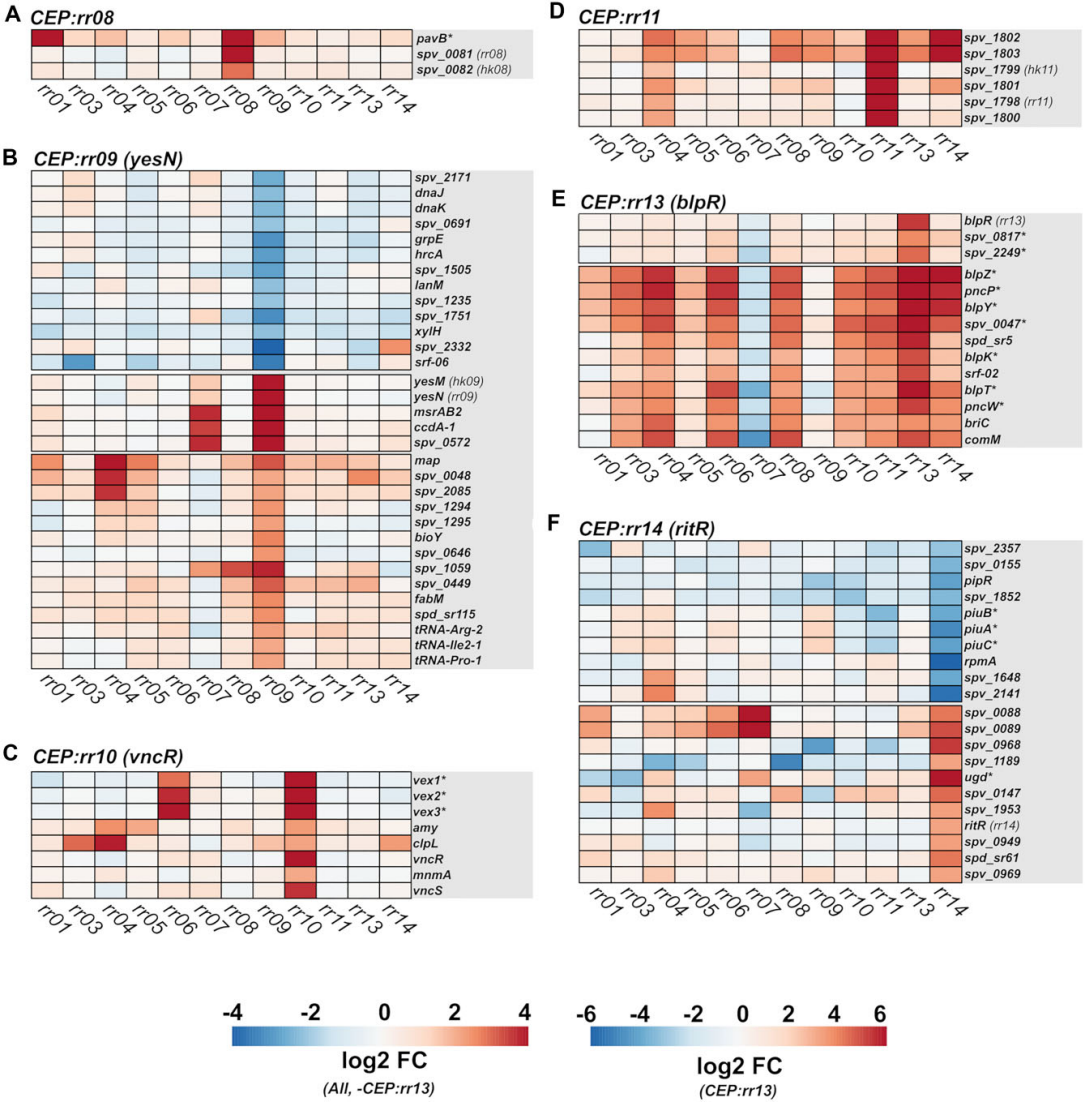
Identification of TCS regulons by RR overexpression – part 2. Clustered heatmaps of RR-specific differentially expressed genes (|logFC|>2, FDR < 0.05) in RR overexpression mutants ((**A**) CEP::*rr08*, (**B**) CEP::*rr09*, (**C**) CEP::*rr10*, (**D**) CEP::*rr11*, (**E**) CEP::*rr13*, (**F**) CEP::*rr14*). Previously identified TCS regulon genes are highlighted by an asterisk (*). Please note that in the CEP:*rr13* (blpR) heatmap, a cluster of known target genes with less specificity is included. The broad induction of BlpR target genes is likely attributed to ComE co-activation.

With this approach, RR-specific differentially expressed genes were identified for all RR overexpression mutants. Clusters of the most strongly differentially expressed genes (|logFC| > 2, FDR < 0.05), with a high degree of RR-specificity, based on evaluation of the hierarchically clustered heatmaps, are presented in Figures 6 and 7. In most cases, both the panGWAS and RNA-seq data validated previously identified TCS regulon members (marked by an asterisk in the heatmaps). However, several new members were also identified. These included *ptvRABC* for VraSR, several phosphotransferase system (PTS) operons (e.g. *bguADBC*, and *spv_0425-…-lacE-1*) and genes involved with nicotinamide adenine dinucleotide (NAD^+^) biosynthesis/transportation (*nadC* and *pnuC*) for PnpRS, and the *ccdA-1-spv_0572-msrAB2* operon for YesNM (Figures 6 and 7, and Supplementary Table S6).

For the *ciaR* overexpression mutant (CEP::*rr05*), 18 CiaR-specific differentially expressed genes were identified. Most of these were involved with carbohydrate utilization (e.g. *glpKOF*, *fruRBA*, and *celRCD*), and have not previously been described as members of the CiaR-regulon. In fact, most of the previously identified CiaR regulon genes were not identified as differentially expressed using the |log_2_ (FC)| > 2 cut-off, except for the *manLMN* operon. A heatmap of known CiaR-regulated genes revealed that some of the well-known members of the CiaR regulon (*htrA-parB* and *malQP*) were weakly induced in the CEP::*rr05* strain, however, not significantly (FDR > 0.05) (Supplementary Figure S3). An examination of the upstream sequences for the newly identified CiaR-regulated genes (*celA*, *fruR*, *glpK* and *spv_1585*) did not reveal any strong CiaR binding motifs in the vicinity of their promoter sequences (Supplementary Figure S3). We suspect the observed induction of these genes to be an indirect effect, possibly caused by CiaR-mediated downregulation of the carbohydrate transporter system ManLMN, which is a central metabolic regulator involved with carbohydrate utilization (51).

VncRS TCS was reported to regulate the *vex123* operon (52), which is supported by our data (Figure 7C). Interestingly, however, our data also revealed a strong induction of this operon by CbpR, suggesting a possible overlap in the target regulon of CbpRS and VncRS. TCS11 is among the least studied pneumococcal TCSs. One study identified several genes involved with sugar utilization as being downregulated in a *rr11* deletion mutant (53). We were only able to identify the *spv_1798–1803* operon as the only specific target of RR11, both by RNA-seq and predicted by panGWAS. This operon encodes TCS11 and 4 hypothetical proteins, including two ABC transporters of unknown function (SPV_1800 and SPV _1801). Consistent with previous findings, both the *piu* operon (encoding an iron transporter system) and the *ugd* gene (encoding the UDP-glucose 6-dehydrogenase) was regulated by RitR. However, inconsistent with previous findings, *ugd* was activated, and not repressed by RitR overexpression (54).

### Evaluation of upstream sequences of RR-regulated genes reveals candidate RR binding motifs

**Figure 8.**
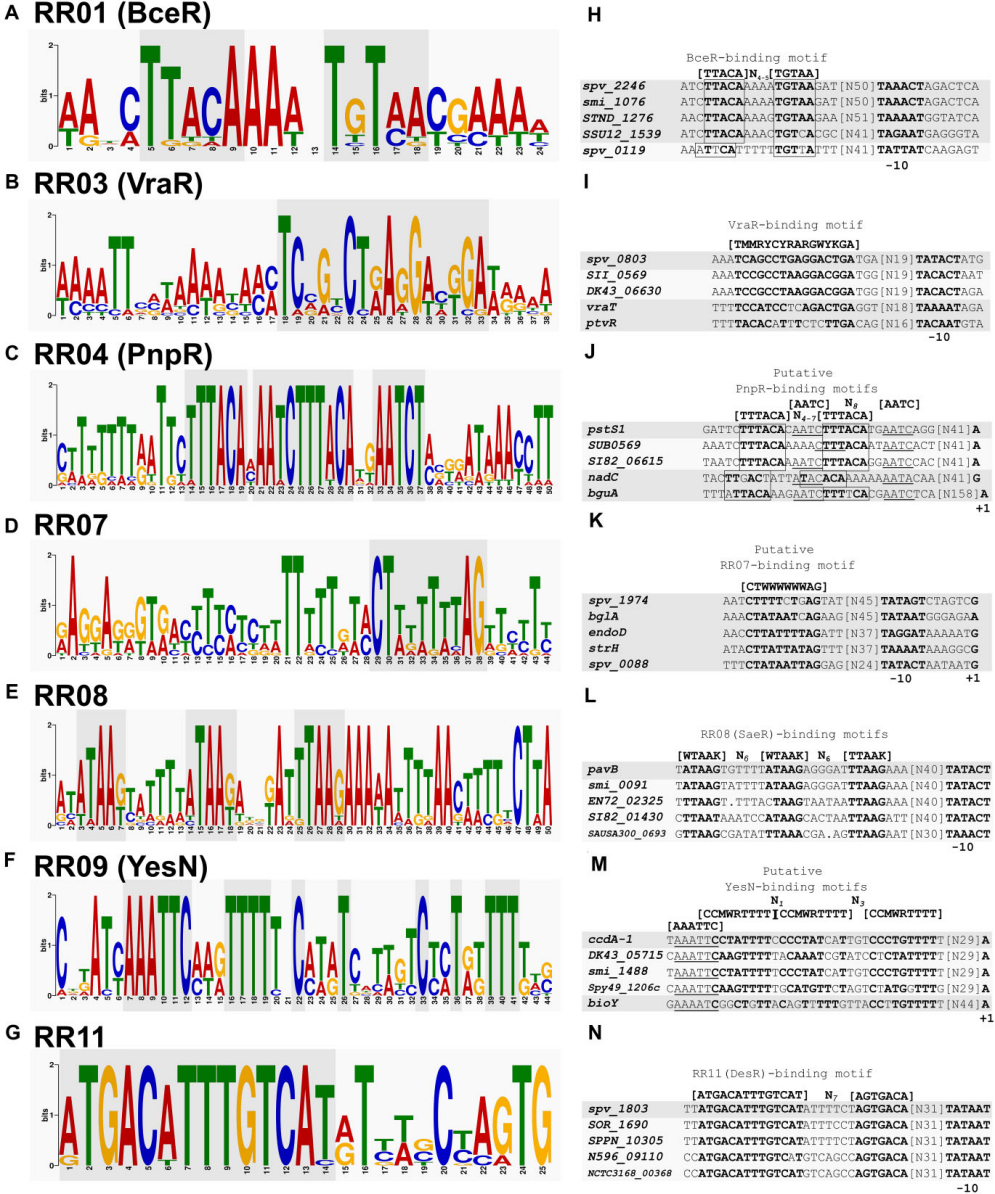
DNA-binding motif discovery of pneumococcal response regulators with GLAM2. Candidate DNA-binding motifs were discovered for (**A**) BceR, (**B**) VraR, (**C**) PnpR, (**D**) RR07, (**E**) RR08, (**F**) YesN and (**G**) RR11. For identification of conserved motifs, the upstream region of the most strongly induced genes (*bceAB*, *spv_0803/vraT*, *pstS1*, *endoD*, *pavB*, *ccdA-1*, *spv_1803*, respectively for VraR, PnpR, RR07, RR08, YesN and RR11) together with at least 10 homologous sequences from other streptococcal and non-streptococcal species, also harboring the respective TCS homologue were used for the identification. (**H–N**) Putative RR binding motifs in promoter regions of pneumococcal RR target genes (dark grey) aligned with putative motifs identified in homologous upstream regions from other species (light grey).

The DNA binding motifs of several pneumococcal RRs are still unknown. To identify putative binding motifs, the motif discovery tool GLAM2 was used. As input for GLAM2, upstream sequences (∼300 nt) of the most highly induced RR target genes from pneumococcus, together with upstream sequences of homologues genes from other streptococcal species that also possess the TCS homologue, were used. Sequences used as input are presented in Supplementary Table S7. Conserved repeat sequence patterns present in the generated sequence logos were used for proposing candidate motifs. Using this approach, candidate binding motifs for 7 pneumococcal RRs were identified (Figure 8A–G). These candidate motifs were observed in promoter regions of both previously and newly identified target genes (e.g. *spv_0803* and *ptvRABC* for VraR, and *pstS1* and *nadC* for PnpR) (Figure 8H-N). A candidate VraR binding motif has previously been discovered in pneumococcus (43,55), and a similar motif was also identified here: [TMMRYCTRARGWYKGA]-N_31-34_-TSS (Figure 8B). The remaining candidate motifs have not previously been identified in pneumococcus, but some of them are consistent with motifs for homologous RRs from other species. For example, the candidate motifs for RR01 and RR08 are similar to the motifs identified for BceR from *S. mutans (*[TTACAA]-N_2_-[TTGTAA]) and the RR08 homologue SaeR from *S. aureus* ([GTTAA]-N_6_-[GTTAA]), respectively (Figure 8A, H and E, L) (56,57). Also, the identified motif for RR11 ([ATGACATTTGTCAT]-N_7_-[AGTGACA]) is highly similar to the binding motif of its homologue DesR from *B. subtilis* ([ATGACA]-N_2_-[TGTCAT]) (Figure 8G, N) (58,59). We have previously characterized the regulon of TCS07, but did not identify a binding motif for RR07 (47). Interestingly, the approach applied here revealed a candidate RR07 binding motif using *endoD* upstream sequences: [CTWWWWWWAG]-N_39-60_-TSS (Figure 8C). This motif was also present in promoter regions of other TCS07 regulon genes (Figure 8K). For YesN, a clear repeat sequence motif was not identified. However, the sequence logo did reveal a very conserved [AATTC] sequence, and some very conserved cytosines and thymine stretches that could constitute parts of a DR sequence motif as presented in Figure 8F, M. Using FIMO with the GLAM2-predicted candidate motifs to scan upstream regions of all RR-specific differentially expressed operons indicated that the motifs were present in 61% (RR07 (22/36)) to 100% (RR01 (6/6), RR08 (2/2), RR11(1/1)) of the upstream operon regions (Supplementary Figure S4, Supplementary Table S8).

### Several sRNAs show RR-specific expression patterns

**Figure 9.**
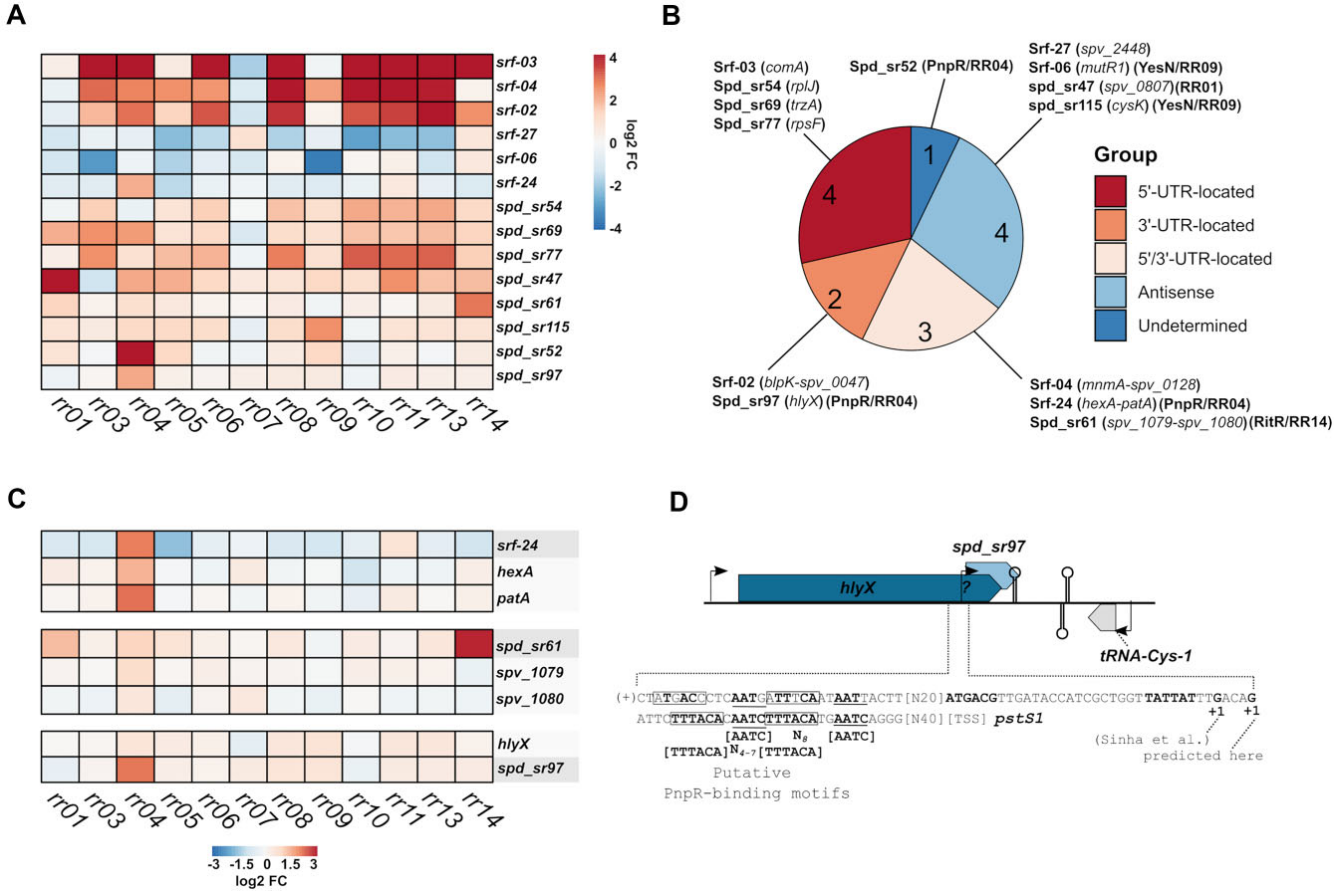
Differential expression of small non-coding RNAs (sRNAs) in RR overexpression mutants. (**A**) Heatmap of 14 differentially expressed sRNAs. (**B**) Differentially expressed sRNAs were divided into specific groups depending on their location, predicted from previous studies (61) and resources available online (pneumobrowse (60)). For UTR-located and antisense sRNAs, their associated genes are specified in parenthesis. RR-specificity is also highlighted in a parenthesis. (**C**) Heatmap of selected UTR-located sRNAs and their associated genes. (**D**) Genomic location of the sRNA Spd_sr97 with a putative PnpR-binding motif.

TCSs control an expanded network of genes indirectly via controlling the expression of *trans*-acting regulatory sRNAs. In pneumococcus, only CiaRH is known to control the expression of sRNAs (i.e. the csRNAs). To identify novel TCS-regulated sRNAs, we examined the expression of 30 sRNAs annotated in D39V (60), and 74 sRNAs recently annotated in D39W (61). In total, 14 sRNAs were differentially expressed among the 12 RR overexpression mutants investigated in this study, but only a subset showed RR-specific expression patterns (Figure 9A). Several of the differentially expressed sRNAs are not validated as primary transcripts and are predicted to reside within untranslated regions (UTRs), suggesting that they are co-transcribed with their cognate mRNA. Notably, nine out of 14 differentially expressed sRNAs were predicted to exist within UTRs (Figure 9B). The remaining five were four antisense encoded sRNAs, and one was of an undetermined origin.

Seven differentially regulated sRNAs show clear RR-specific expression patterns, including Spd_sr47 (RR01-specific), Srf-24, Spd_sr52, Spd_sr97 (all PnpR-specific), Srf-06, Spd_sr115 (all YesN-specific) and Spd_sr61 (RitR-specific). Some of these are UTR-located sRNAs (Srf-24, Spd_sr61 and Spd_sr97). For Srf-24, the expression pattern coincided with the expression of the associated gene *patA* (Figure 9C). For Spd_sr61 and Spd_sr97, induction did not coincide with induction of the associated genes (*spv_1079*/*spv_1080* and *hlyX*, respectively), and thus might be independently regulated by the RRs. Spd_sr97 is located within the *hlyX*-3′-end and shares a transcriptional terminator with *hlyX* (Figure 9D). It was discovered as a processed transcript (61), and is possibly co-transcribed with the *hlyX* mRNA. However, with the identification of a predicted promoter region and a weak putative PnpR binding motif upstream the predicted Spd_sr97 transcription start site (TSS) (Figure 9D), it cannot be ruled out that Spd_sr97 is independently transcribed from the *hlyX* gene and regulated by PnpR.

Three antisense sRNAs (Srf-06, Spd_sr47 and Spd_sr115) also show RR-specific expression patterns. However, while the sRNAs were strongly regulated by either YesN or RR01, we were not able to identify putative binding motifs in the vicinity of their promoter regions.

## Discussion

Mapping the main bacterial sensory systems governed by TCSs, is key to understanding bacterial pathogenesis and virulence. This study uncovers novel details on pneumococcal TCS regulatory networks through a comprehensive analysis of multiple pneumococcal TCSs using a panGWAS-based co-occurrence analysis, transcriptomic analyses, and motif discovery of candidate RR binding motifs. Our finding that all TCSs are present across all pneumococcus isolates, and some are universal across the entire streptococcal genus, underscores their significance. Notably, we identified TCS01, VicRK, CiaRH, BlpHR and RitR in the non-pathogenic *S. thermophilus*. This presence in a non-pathogenic strain suggests a broader functional role for these TCSs beyond virulence in pathogenic contexts (62).

Identification of RR target genes through mutagenesis and overexpression can present challenges, as RR activity relies not only on the level of expression but also on the state of phosphorylation. While some studies have effectively used phosphomimetics (63), it is noteworthy that that while phosphorylation typically promotes subunit oligomerization and DNA binding, exceptions do exist (64). Our experience has shown that overexpressing native RRs in conjunction with RNA-sequencing reliably identifies RR targets; findings later confirmed by other studies (47,65). Consequently, the same methodology was adopted for this study. Our data validated previous findings on the regulons of TCS01 (66), VraRS (55), PnpRS (67), CbpRS (68), VncRS (52), BlpRH (29) and RitR (54). However, novel candidate regulon members were also identified. For TCS01, the virulence gene *pavB* and the RtgR/S-regulated *rtg* operon were among newly identified candidate members of its regulon. Both *pavB* and the RtgR/S system contribute to nasopharyngeal colonization (50,69). Since AMP resistance, mediated by the TCS01-regulated BceAB transporter, also provide an important advantage during host colonization, it emphasizes a putative role for TCS01 in mediating colonization. Most of the differentially expressed genes in CEP::*rr01*, except for *spv_0805* (*bceB*) (Figure 8), were not identified as co-occurring with TCS01 across the streptococcal clade. This could indicate that the regulon of TCS01, and its function, is species dependent.

Multiple novel candidate regulon members were also discovered for PnpRS, the pneumococcal regulator of the Pho regulon (70). Although a previous study has reported TIGR4-specific regulation of the *psaBCA* operon (71), the only well-characterized member of the PnpR regulon, so far, is the *pst1* operon (67). This is corroborated with the *pstS1* and *pstA1* genes being highly conserved across streptococci with PnpRS (Figure 5). Indeed, our RNA-seq data demonstrated high induction of the *pst1* operon in the CEP::*rr04* strain, while no regulation of *psaBCA* was observed. Instead, several genes involved with NAD^+^ biosynthesis, PTSs, and efflux transporters were strongly induced in the CEP::*rr04* strain (Figure 6B and Supplementary Table S6). In particular, the NAD^+^ biosynthesis genes, *pnuC* and *nadC* were highly induced in the CEP::*rr04* strain, suggesting a strong link between NAD^+^ biosynthesis and management of P_i_ levels. Only a few studies have previously reported at similar link between the Pho regulon and NAD^+^ biosynthesis (72,73).

Curiously, the most highly induced genes in the *ciaR* overexpression strain were involved with carbohydrate utilization (e.g. *glpKOF*, *fruRBA* and *celRCD*), and not previously characterized CiaR regulon members. We hypothesize that the observed induction of these genes is an indirect effect caused by CiaR-mediated downregulation of the carbohydrate transporter system ManLMN, which is a transporter of multiple carbohydrate substrates and a central metabolic regulator of carbohydrate utilization (51). It remains unclear why multiple CiaR regulon members were only weakly induced (*htrA-parB*, *malQP*, *ccnB* and *ccnD*), while others were not induced at all (*tarIJ-licABC*, *dltXABCD*, *ccnA*, *ccnC* and *ccnE*). However, we notice that a similar picture was observed by Slager et al., who investigated activation of CiaR regulon genes during competence (43). In their study, a handful of non-induced CiaR regulon genes from our study (*tarIJ-licABC*, *ccnA*, *ccnC* and *ccnE*) were unaffected during competence despite an activation of the remaining CiaR regulon. This might question whether these genes are legitimate members of the CiaR regulon. However, this is refuted by several other studies reporting CiaR-dependent regulation, and the existence of strong CiaR-binding motifs in the promoter region of these genes (25,74). In addition, we do not observe a *comDE* upregulation in the *ciaR* overexpressing mutant. The fact that several well-characterized CiaR-regulated genes are only weakly induced or unaffected by *ciaR* overexpression, could indicate that other factors are required for complete CiaR activation, such as a state of phosphorylation not captured during RR overexpression.

YesNM has remained largely uncharacterized. Early studies reported strain-dependent regulation by YesN (35), and more recent data suggest that YesNM is involved with carbohydrate metabolism (34). In the latter study, the findings were primarily based on transcriptional changes observed in a HK (*yesM*)-deficient strain, as limited changes were observed in a RR (*yesN*)-deficient strain. Our data did not reveal similar findings regarding carbohydrate metabolism. Instead, the most notable observation in the *yesN* overexpression mutant was a high induction of the *ccdA-1-spv_0572-msrAB2* operon, which is involved with extracellular oxidative stress management (75). Furthermore, the *ccdA-1* and *spv_0572* genes showed significant co-occurrence with *yesNM* across all streptococcal species analyzed (Figure 5). We cannot rule out that YesN works as a repressor of a group of genes in the absence of its cognate HK in its non-phosphorylated state, as speculated by Hirschmann *et al.*, which could explain the differences observed in the *yesM* mutant (34). However, it is also possible that some of the responses observed in the *yesM* deficient mutant by Hirschmann *et al.* could be indirect effects caused by disrupted HK crosstalk with other regulatory proteins.

TCS cross-regulation is a common mechanism used for diversifying and coordinating a response to a single stimulus. In our study, cross-regulatory events were also observed. For instance, overexpression of *rr01* lead to weak induction of *vraRS* and *ciaRH* transcription, including their regulons, and overexpression of *pnpR* and *rr07* lead to weak induction of *TCS11* and *YesNM*, respectively. TCS01, VraRS and CiaRH are all involved with aspects of cell envelope stress, which might be the functional link explaining the observed cross-regulatory relationship (55,66,76). In other cases, an overlap in TCS regulons was observed without cross-activation of their respective TCSs. This was the case for the closely related CbpRS and VncRS, which both strongly induced expression of the *vex123* operon without transcriptional TCS cross-activation. Both TCSs were predicted to strongly co-occur with the *vex1* and *vex3* genes and were the only TCSs so similar that they were indistinguishable from each other across the streptococcal clade on a sequence level. As none of the remaining TCS regulon genes (e.g. *cbpA* for CbpRS, and *mnmA* for VncRS) were induced across both RR overexpression mutants, we assume *vex123* induction is not a result of HK activation of their non-cognate RR either. Instead, it is most likely that the *vex123* promoter region contain RR binding motifs for both CbpR and VncR, although these motifs are currently unknown.

Using the GLAM2 motif discovery tool, we were able to identify promising candidate RR binding motifs for a handful of the pneumococcal RRs. Some of these supported previous findings (e.g. VraR), while others were novel (e.g. PnpR, RR07 and YesN). For RR08 and RR11 we were able to identify motifs that were very similar to the binding motifs of homologous RRs from other organisms (SaeR in *S. aureus*, and DesR in *B. subtilis*, respectively) (56,58). While the validation of previously identified RR binding motifs demonstrates the robustness of this approach, biochemical assays are still needed to validate these candidate RR binding motifs.

TCS regulatory networks are often modulated by regulatory sRNAs, and multiple TCSs also induce transcription of their own set of *trans*-acting sRNAs, including CiaRH, which is the only pneumococcal TCS known to induce expression of sRNAs (17). In this study, we evaluated if any additional sRNAs displayed RR-specific expression patterns. 14 out of >100 annotated sRNAs were differentially expressed among the investigated RR overexpression strains, and seven of these showed clear RR-specific expression patterns. In addition to these, several sRNAs showed significant upregulation across multiple RR overexpression strains. These are most likely competence-induced sRNAs, indicating that the competence response could include multiple layers of RNA-mediated regulation, in addition to the one mediated by the csRNAs (77). Except for evaluating the presence of potential candidate RR binding motifs in their promoter regions, no further validation or characterization was performed in this study. We did notice a general enrichment of predicted UTR-derived sRNAs among the differentially expressed sRNAs, which is a noteworthy observation considering the increased appreciation of the importance of UTR-derived RNAs in bacterial regulatory mechanisms, reported in recent years (78,79).

The panGWAS approach showed that the TCSs are highly conserved, not only in pneumococcus, but across the entire streptococcal clade. Due to the conserved nature of the TCSs, we were compelled to expand the analysis to include the other streptococcal species, increasing the diversity of the gene pool. The increased diversity meant that the sequence identity threshold for identifying genes had to be lowered and thus reduced the sensitivity while retaining the specificity. Using the entire streptococcal clade in the panGWAS also meant that any identified co-occurring genes would be associated with the given TCS in all streptococcal species, thus ruling out any species-specific genes. However, the method still proved effective and showed concordance with the RNA-seq analysis in most cases.

In conclusion, this study revealed extensive insights into the regulatory network governed by multiple pneumococcal TCSs. In addition to comprehensively mapping pneumococcal TCS regulons, this study also detected TCS cross- and autoregulatory events and identified novel candidate RR binding motifs. By using a panGWAS-based approach to identify TCS co-occurring genes, we also demonstrated that this approach is a useful tool for preliminary identification of regulatory targets, as highly induced target genes also strongly co-occurred with their TCS. In the future, this approach could prove useful as a complementary tool to transcriptomics- or proteomics-based approaches for evaluating potential targets of other regulatory factors.

## Supplementary Material

lqae039_Supplemental_Files

## Data Availability

The data underlying this article are available in NCBI’s Gene Expression Omnibus (74) and are accessible through GEO Series accession number GSE225902 (https://www.ncbi.nlm.nih.gov/geo/query/acc.cgi?acc=GSE225902).
