# Supplementary material for: A comprehensive analysis of pneumococcal two-component system regulatory networks
Source: NAR Genom Bioinform. 2024 Apr 22;6(2):lqae039. doi: 10.1093/nargab/lqae039 (PMC11034029; doi:10.1093/nargab/lqae039)
Supplement: lqae039_Supplemental_Files [file lqae039_supplemental_files.zip › 01_03_24_A_comprehensive_analysis_NAR_Genomics_&_Bioinformatics_Supplementary_material_revision_JSP.pdf]

SUPPLEMENTARY FIGURES AND TABLES

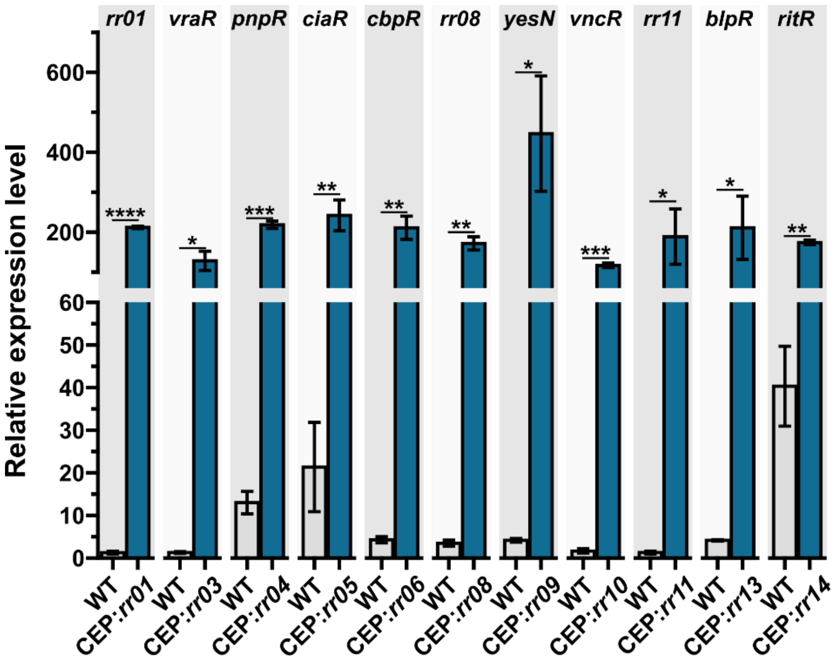

Figure S1. Chromosomal overexpression of pneumococcal RRs. Transcriptional overexpression of response regulators (RR) from the chromosomal expression platform (CEP) site in D39V was validated by RT-qPCR. RT-qPCR was performed on RNA harvested from the specified strains grown in C+Y medium at 37°C to an OD_{600} 0.4, in biological triplicates. Bar charts represent mean relative gene expression levels normalized to *gyrA* mRNA, with error bars representing standard deviations and asterisk depicts level of statistical significance (Student's t-test. *: p < 0.05, **: p < 0.01, ***: p < 0.001).

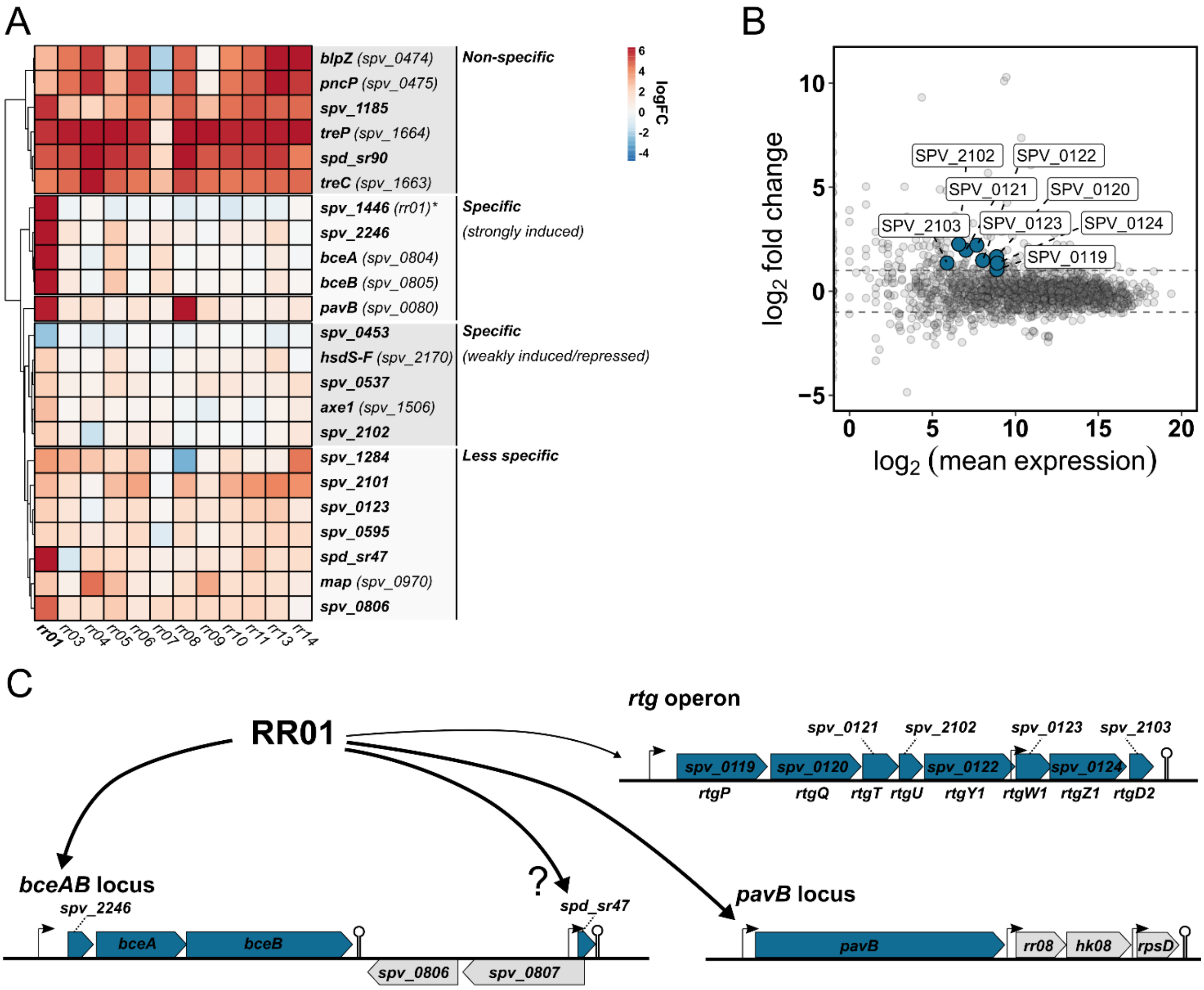

Figure S2. Identification of RR01 (BceR)-regulated genes. (A) Clustered heatmaps of 23 differentially expressed genes from CEP::*rr01*. **(B)** MA plot depicting fold change levels in CEP::*rr01* versus the wild-type. Genes from the *rtg* operon are highlighted in blue. **(C)** Summary of identified RR01-regulated genes.

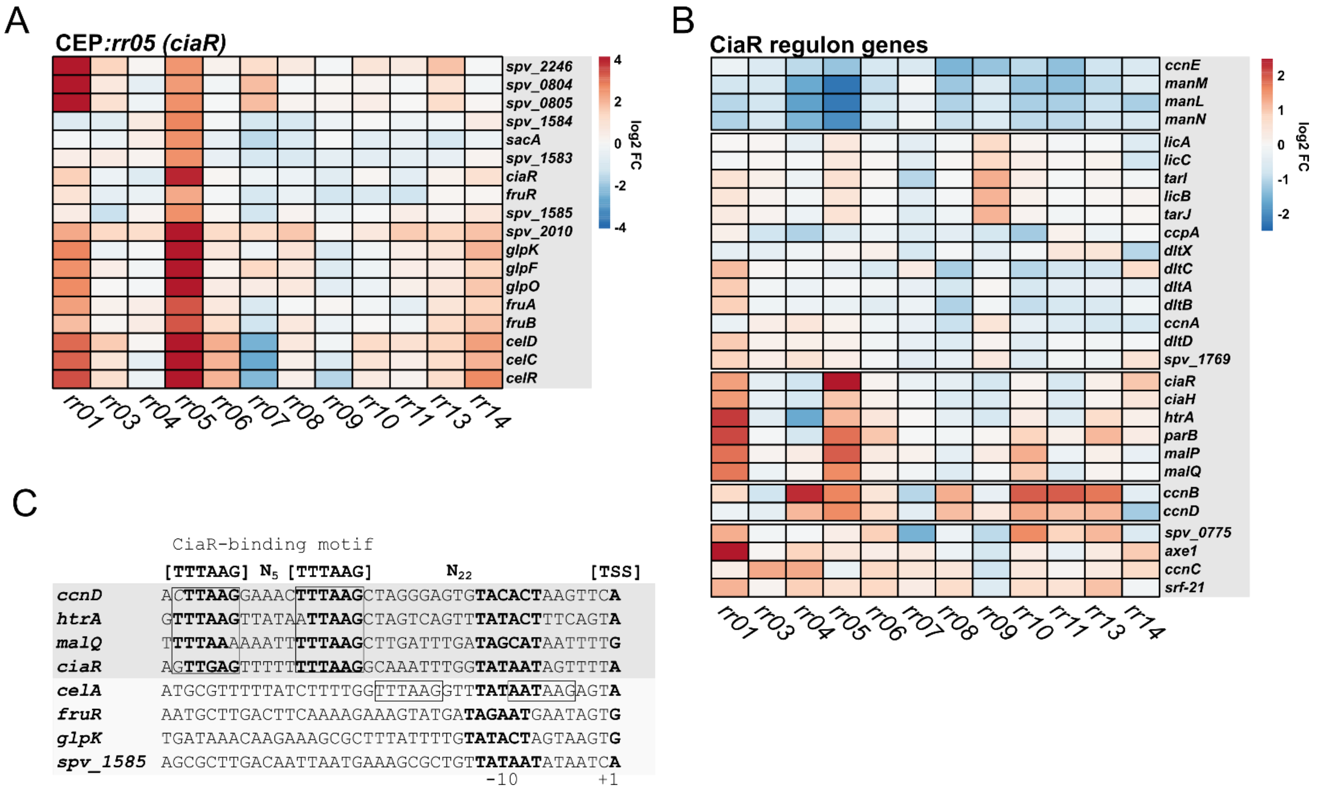

Figure S3. Identification of CiaR-regulated genes. **(A)** Heatmaps of the most CiaR-specific regulated genes according to a clustered heatmap analysis, identified by *ciaR* overexpression. **(B)** Heatmap of expression fold-change levels of previously identified CiaR-regulated genes. **(C)** Previously identified CiaR-binding motifs upstream CiaR-regulated genes aligned with upstream sequences of newly identified CiaR-regulated genes.

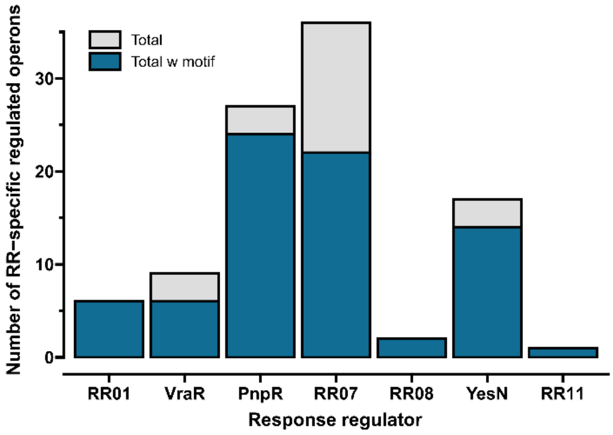

Figure S4. Total number of RR-specific regulated operons with candidate RR binding motifs. Upstream sequences of operons being differentially expressed in RR overexpression mutants (CEP:*rr01* (RR01), CEP:*rr03* (VraR), CEP:*rr04* (PnpR), CEP:*rr08* (RR08), CEP:*rr09* (YesN), and CEP:*rr11* (RR11)), in a RR-specific manner, were scanned for the presence of the GLAM2 predicted candidate motifs.

Table S1. PCR primers used in study.

Sequence (5'->3')	Name	#
AGTTAAAGAACTTGGACTGCGC	*CEP_up_F*	1
TAATTTTCCTCCTATTTAGATCTTGCATGTATAG	*CEP_up_R*	2
TAAGGATCCCTCCAGTAACTCG	*CEP_down_F*	3
CAATGTGAAAGCGATCAAGAACG	*CEP_down_R*	4
CTATACATGCAAGATCTAAATAGGAGGAAAATTAATGCACAAG ATTTTATTAATAGAAGATGATCAG	*RR01_F*	5
CGAGTTACTGGAGGGATCCTTATCAAGCATGCTTCAATCCGTA CC	*RR01_R*	6
CTATACATGCAAGATCTAAATAGGAGGAAAATTAATGAAAAAA ATACTAATTGTAGATGATGAGAAAC	*RR02_F*	7
CGAGTTACTGGAGGGATCCTTATCAAGCATTATTTCTCATGTA ATACCCTAC	*RR02_R*	8
CTATACATGCAAGATCTAAATAGGAGGAAAATTAATGAAAATT TTACTAGTAGATGACCATGAAATG	*RR03_F*	9
CGAGTTACTGGAGGGATCCTTACTAAAACTCCTCTTGCCCCAC	*RR03_R*	10
CTATACATGCAAGATCTAAATAGGAGGAAAATTAATGACAAAA CAAGTCTTATTAGTGGATG	*RR04_F*	11
CGAGTTACTGGAGGGATCCTTACTATAACTCCTTGAACTTATA ACCATAACCC	*RR04_R*	12
CTATACATGCAAGATCTAAATAGGAGGAAAATTAATGATAAAA ATCTTATTGGTTGAGGATGAC	*RR05_F*	13
CGAGTTACTGGAGGGATCCTTATTACTGAACATCTTTTAAAAG ATACCCAAC	*RR05_R*	14
CTATACATGCAAGATCTAAATAGGAGGAAAATTAATGAACATT TTAGTTGCAGATGACG	*RR06_F*	15
CGAGTTACTGGAGGGATCCTTATCATAAGCTAATCTTATACCC AACATTTTTC	*RR06_R*	16
CTATACATGCAAGATCTAAATAGGAGGAAAATTAATGGGAAAG ACAATTTTACTCGTTG	*RR08_F*	17
CGAGTTACTGGAGGGATCCTTATCATGTTTGTCCTCTCG GTTTC	*RR08_R*	18
CTATACATGCAAGATCTAAATAGGAGGAAAATTAATGAC CTACACAATCTTAATCGTAGAAG	*RR09_F*	19
CGAGTTACTGGAGGGATCCTTATCATCGGTCTTCTCCCTTCTT AAAC	*RR09_R*	20
CTATACATGCAAGATCTAAATAGGAGGAAAATTAATGAAAATT TTAATTGTAGAAGATGAAGAGATGATC	*RR10_F*	21
CGAGTTACTGGAGGGATCCTTATCATTTTCGCTCCAATTTATA ACCAAC	*RR10_R*	22
CTATACATGCAAGATCTAAATAGGAGGAAAATTAATGAAAGTA TTAGTCGCAGAAGATC	*RR11_F*	23
CGAGTTACTGGAGGGATCCTTATCATAACCAACCAGATTCTTT TGCG	*RR11_R*	24
CTATACATGCAAGATCTAAATAGGAGGAAAATTAATGAGAATA TTTGTTTTAGAAGATGATTTTTCCC	*RR13_F*	25
CGAGTTACTGGAGGGATCCTTATCAGTGTAAGTTATTAATAGC CTCAGAC	*RR13_R*	26
CTATACATGCAAGATCTAAATAGGAGGAAAATTAATGGGGAAA CGGATTTTATTACTTG	*RR14_F*	27
CGAGTTACTGGAGGGATCCTTACTATTCTTGCATGGTATATCC AACAC	*RR14_R*	28
GGGATTTGAAGTGGTCCTGG	*rr01_qPCR_F*	29
ATGATAGGTACCTTGGAAATCTTGC	*rr01_qPCR_R*	30

CAATTTGAAGCAGAGCAACCAG	*rr02_qPCR_F*	31
CCCCAAGTTCCAAACCGATAAC	*rr02_qPCR_R*	32
CCATGAAATGGTCCGTTTGG	*rr03_qPCR_F*	33
GCATGACAATATCCATGACAATGAC	*rr03_qPCR_R*	34
TTGGTAACAAATGGACGGAAGG	*rr04_qPCR_F*	35
CTCAGCCGCTTACAAACTTCC	*rr04_qPCR_R*	36
TGATGGAGAAGAAGGTCTCTACG	*rr05_qPCR_F*	37
AGTCATAATCAGAACTGGTGTCG	*rr05_qPCR_R*	38
AGCATTTCTGACAGAAGAGGG	*rr06_qPCR_F*	39
AAACCACTCTTCCTAGGCATC	*rr06_qPCR_R*	40
TGGTGACAGAAGGTCTGAAGC	*rr07_qPCR_F*	41
GACATCGGAAATGATGACATCG	*rr07_qPCR_R*	42
TCTTGGTAGCCCATGATGGAC	*rr08_qPCR_F*	43
AAATAGGAAAGGCTGCTCTGG	*rr08_qPCR_R*	44
GGGAATTGATCCAAAAGCAGG	*rr09_qPCR_F*	45
TTTGAGCAGGTAGTCGTCCAC	*rr09_qPCR_R*	46
TGAAGAGATGATCCGTGAGGG	*rr10_qPCR_F*	47
ACTTCTAAGCCGTTAAGCTTGG	*rr10_qPCR_R*	48
CAAAGTATGTTGCGAGATGCC	*rr11_qPCR_F*	49
AGGCATTTCTACGTCAAGGATG	*rr11_qPCR_R*	50
ATTGAGAAAAAGGGATTTGAAGTGG	*rr12_qPCR_F*	51
CCTTATCAACAAAATCTAGGGCTG	*rr12_qPCR_R*	52
ATTCGAAATGAGGAGATGAAGGG	*rr13_qPCR_F*	53
AAACTCCTCTGCCGAAAGG	*rr13_qPCR_R*	54
TTTGGAACTCCAGAAAGAGCAG	*rr14_qPCR_F*	55
TGTTTGTCGGTGAACGCTCTC	*CEP_seq_F*	56
GAGCAATCTTTTGGTTGATGATACGA	*CEP_seq_R*	57

Table S2. Strains used in study.

Strain (name)	Organism	Genotype	Reference
D39V (*wild-type*)	*S. pneumoniae* D39V		(Slager J et al., 2018)
D39V CEP:*rr01*	*S. pneumoniae* D39V	D39V; *CEP::[spec:P3$_{con}$-rr01]*	This study
D39V CEP:*rr03 (vraR)*	*S. pneumoniae* D39V	D39V; *CEP::[spec:P3$_{con}$-rr03]*	This study
D39V CEP:*rr04 (pnpR)*	*S. pneumoniae* D39V	D39V; *CEP::[spec:P3$_{con}$-rr04]*	This study
D39V CEP:*rr05 (ciaR)*	*S. pneumoniae* D39V	D39V; *CEP::[spec:P3$_{con}$-rr05]*	This study
D39V CEP:*rr06 (cbpR)*	*S. pneumoniae* D39V	D39V; *CEP::[spec:P3$_{con}$-rr06]*	This study
D39V CEP:*rr08*	*S. pneumoniae* D39V	D39V; *CEP::[spec:P3$_{con}$-rr08]*	This study
D39V CEP:*rr09 (yesN)*	*S. pneumoniae* D39V	D39V; *CEP::[spec:P3$_{con}$-rr09]*	This study
D39V CEP:*rr10 (vncR)*	*S. pneumoniae* D39V	D39V; *CEP::[spec:P3$_{con}$-rr10]*	This study
D39V CEP:*rr11*	*S. pneumoniae* D39V	D39V; *CEP::[spec:P3$_{con}$-rr11]*	This study
D39V CEP:*rr13 (blpR)*	*S. pneumoniae* D39V	D39V; *CEP::[spec:P3$_{con}$-rr13]*	This study
D39V CEP:*rr14 (ritR)*	*S. pneumoniae* D39V	D39V; *CEP::[spec:P3$_{con}$-rr14]*	This study

Table S3. TCS trait matrix (Supplementary file). Occurrence (1) and absence (0) of pneumococcal TCS within genomes of the *Streptococcus* genus.

Table S4. Full list of genes significantly co-occurring with pneumococcal TCSs identified by panGWAS (Supplementary file).

Table S5. All differentially expressed genes in RR overexpression mutants (Supplementary file). Raw read counts, logFC, and FDR values for each gene in each response regulator overexpression mutant (RR01-RR14). Up- and downregulated (logFC >2 and logFC <-2) genes in each mutant are color coded green and red, respectively. A blue color indicates a significant FDR value (<0.05).

Table S6. Overview of RR-regulated genes. Table summarizes TCS-specific differentially expressed genes (|log2 (FC)| >2, FDR<0.05) from the different RR overexpression mutants. TCS-specificity was determined based on the clustered heatmaps analysis (see Figure 5 and 6). The list also includes direction of regulation (up / down), strength of regulation (+++: |log2 (FC)| >3, ++: |log2 (FC)| >2, +: |log2 (FC)| >1) and known biological function (n.a.: no available information).

Response regulator	Regulated genes	Direction	Strength	Biological function
**RR01**	*spv_2246-bceAB*	Up	+++	Bacitracin transport
	pavB	Up	+++	Adhesin
	spv_0119-…-spv_2103 (*rtg* operon)	Up	+	Unknown, encodes GG peptides
**VraR**	*spv_0355-cbpGK*	Up	+++	Choline-binding proteins
	spv_0803	Up	+++	Phage shock protein
	vraTSR-alkD	Up	+++	TCS operon
	ptvRABC	Up	+	Vancomycin tolerance
	clpL	Up	+	Protein chaperone
	spxA2	Up	+	Transcriptional regulator
**PnpR**	*pstSCAB1-phoU1*	Up	+++	Inorganic phosphate transport
	nadC-…-spv_1824	Up	+++	NAD(+) biosynthesis (NiaR-regulated)
	pnuC	Up	+++	NAD(+) transport (NiaR-regulated)
	spv_1828	Up	++	n.a.
	bguADBC	Up	++	PTS (cellobiose)
	spv_0293-…-spv_2141	Up	++	PTS (hyaluronan disaccharide)
	spv_2441-spv_1974	Up	++	Glycan metabolism
	spv_1648	Up	++	UDP-glucose 4-epimerase
	spv_1441	Up	++	n.a.
	patAB	Up	++	Multidrug efflux pump
	Srf-24	Up	++	n.a.
	ulaABCDEF	Up	++	PTS (ascorbate)
	spv_0846	Up	++	ABC-transporter
	spv_0162-spv_0163	Up	++	n.a.
	spv_0097	Up	++	Putative cyanate transporter
	spv_2121	Up	++	n.a.
	spv_1827	Up	++	Malonate permease (NiaR-regulated)
	spv_0338	Up	++	n.a.
	dinF-lytA	Up	++	Multidrug efflux transporter and cell wall amidase
	spv_0425-…-lacE-1	Up	++	PTS (lactose)
	spv_1951	Up	++	Macrolide-efflux protein
	hipO-spv_1921	Up	+	n.a.
	spv_0995	Down	++	n.a.
**CiaR**	*glpKOF-spv_2010*	Up	+++	Glycerol utilization
	fruRBA	Up	+++	Fructose utilization
	celRC-spv_0282-celD	Up	+++	Cellobiose utilization

	Spv_1585-…-sacA	Up	++	Fructooligosaccharide (FOS) utilization
	spv_2246-spv_0804-spv_0805	Up	++	Bacitracin transport
	htrA-parB	Up	+	Protein degradation and chromosome segregation
	malQP	Up	+	Glycogen degradation
	ccnB, ccnD	Up	+	Regulatory RNAs
	manLMN	Down	++	PTS (multiple sugars)
**CbpR**	*cbpA*	Up	+++	Choline-binding protein (virulence)
	vex123	Up	++	FtsX-like ABC-transporter
**RR08**	*pavB*	Up	+++	Adhesin
	spv_0081-spv_0081	Up	++	TCS08 operon
**YesN**	*ccdA-1-spv_0572-msrAB2*	Up	**+++**	Oxidative stress resistance
	yesNM	Up	**+++**	
	spv_1294-spv_1295	Up	**++**	n.a.
	bioY	Up	**++**	Biotin uptake
	spv_0646	Up	**++**	Fatty-acid binding protein
	spv_1059	Up	**++**	n.a.
	spv_2332	Down	**+++**	n.a.
	hrcA-…-dnaJ	Down	**++**	Heat-shock genes
	srf-06	Down	**++**	sRNA (antisense to *mutR1*)
	spv_0691	Down	**++**	Transcriptional regulaor
	xylH	Down	**++**	Degradation of aromatic compounds
	*lanM, spv_1751**	Down	***(+)***	Lantibiotic production
**VncR**	*vex123*	Up	+++	FtsX-like ABC-transporter
	vncRS	Up	+++	VncRS operon
	mnmA	Up	++	tRNA modification
	clpL	Up	++	Protein chaperone
	amy	Up	++	α-Amylase
**RR11**	*spv_1798-…-spv_1803*	Up	**+++**	TCS11 operon
**BlpR**	*pncW-blpYZ-pncP*	Up	+++	Bacteriocin immunity
	blpK-spv_0047- srf-02	Up	+++	Bacteriocin production
	blpT	Up	+++	n.a.
	spv_2249-spv_0817	Up	++	CAAX protease
**RitR**	*piuBCDA*	Down	**++**	Iron transport
	spv_1648	Down	**++**	UDP-glucose 4-epimerase
	pipR	Down	**++**	Transcriptional regulator
	spv_2141	Down	**++**	oligohyaluronate lyase
	spv_0155	Down	**++**	ECF-transporter
	spv_1852	Down	**++**	n.a.
	ugd	Up	**++**	UDP-glucose 6-dehydrogenase
	spv_0968-spv_0969	Up	**++**	n.a.
	spv_1953	Up	**++**	n.a.
	spv_0949	Up	**++**	n.a. (part of *rgg0939* operon)
	spd_sr61	Up	**++**	n.a. (sRNA)

Table S7. List of sequences used as input for motif discovery with GLAM2. Between 120-300 bp of the upstream promoter regions were used as input. The organisms from which the sequences are derived are specified by KEGG org codes, and the genes from which they originate are specified by the corresponding D39V name.

RR-of-interest	Corresponding D39V gene	Organisms (KEGG org codes)	Sequence
VraR	*vraT*	spd	CTACACAGTTGAAGAAGTGATTGGCATT<br>GTCCAAGGCTGGAAAGCAGATCTACGCT<br>TGATTATGTGTTCCCTTAACTGTGCCAC<br>CATAGCAGATCTACAAAAAGTAGACTAT<br>CTTCTTTATGGAAAATTAAAAGAAGCAA<br>ATGATCAGATGAAAAAGGCGTAACCAGC<br>GCCTTTTTTTCCATCCTCAGACTGAGGT<br>GACTTTTTTGAATTGTGATAAAATAGAA<br>GGGAGAGGATGAACCT
	vraT	ssi	GAACTCCAGTTCTATCTTCGACTTCGTT<br>TCCTAGGCTACTTTTGATTTTCATTGAG<br>TATTACTTGATAGTATAGACAATATTTA<br>GTTTTATAGCCCTTTAGTTTCTCTTTGA<br>TTACTTCATCAACTTGCTTTGAACTAAA<br>AGTAAACTAAAACTATATATCAGTCAGT<br>CCTATATTTCCTACTTTGGATGGAGTTC<br>TTTTACTTATTTTTTGTTACAATAGTAT<br>AGAAAGGATGGAGAAG
	vraT	sui	GGTTTTATTAAACCGATTTTTCCCTCAC<br>TCTTTTTGCGCTCATTTATATGATAGAG<br>AAGTATTGTTTTTAGTTCCTCAAGTTTG<br>TTCAGTATACTGCTATTAAAAATGTTGG<br>CTAAGAGGCTGGGACTACAGACCAGTAT<br>TGGAAATACTTAGTTTTATTTCGCTGTC<br>TTATTTTCATACCTTAGTCGGAGGAATT<br>TTTGAAAGTTTTTGATACAATAGGATTA<br>GAAAGGTATCGAGACT
	vraT	sans	GTAGTTTTGTTAAATCGATTTTCCCTCC<br>CTCTTTTTTTGCACTCGTTTTACATCAT<br>TGGTAAACATTGCTTCTGCTCAATCAAG<br>TTTATCCAGTATATTGTAATTAAAAATG<br>TTAACTTCTAGGACTACAAACTAGAACT<br>TGAAAATATTTTCTTTCAGTTCACTACC<br>TTCTTTTCATACCTTAGCTGGAGGAATT<br>TTTGTAAGTTTTTGATACAATAGAGTAA<br>GAAAGGTATCGAGGCT
	vraT	sor	CACCATAGCGGATCTGCAAAACGTAGAC<br>TATATTCTTTATGGCAAACTCAAAGAAG<br>CAAAAGATCAGACGTAGAGAGGTCGGGA<br>CAAGAATCCCGCCCTTTTTCTTAGCCTT<br>TTGTCCAACGGTATTGGGCTGAAAGAAC<br>CTAGCTTGTAGGAGCGATTTCTGTCCCA<br>TTGCCTTTTTCCATCCTCAGACCGAGGT<br>GACTTTTTTGAATTGTGATAAAATAGAA<br>GGGAGAGGATGCAGCT

	vraT	smb	CCTACACAGTTGAAGAAGTGATTGGCAT<br>TGTCCAAGGCTGGAAAGACGATCTGCGT<br>TTGATTATGTGTGCCCTTAATTGTGCCA<br>CCATAGCAGATTTGCAAAAAGTAGACTA<br>TCTTCTATATGGAAAATTAAAAGAAGCA<br>AAGGATCAGATGAAAAAGGCGTAACCAC<br>CGCCTTTTTTCCATCTTCAGACCGAGGT<br>GACTTTTTTGAATTGTGATAAAATAGAA<br>GGGAGAGGATGAACCT
	spv_0803	spd	AAGCAGTGGATGGATTAAGGTTTGTACC<br>ATCCTTGCCCAATAGTAACTCTAAACCA<br>CTCAAGCTTTATCGTTGTCAATCCTGCC<br>AGCAAAGGTATCAGCGCAAGCGTAGGAT<br>TGATACCAAACGCTATCGCTGTGGACTT<br>TGTCGAGGTAAATTGCTTCTGATAAATC<br>AGCCTGAGGACTGATGAAAGCCGAGCCT<br>CCCCGTGATATACTATGTCTAACCTAGC<br>AGAAAGAGGAAATATC
	spv_0803	siu	CACCAGGTTTTGATTATCGTTCTACGGT<br>CTTGATTTATCAATGTCAACAATGTCAT<br>ATGTTCTATTTCAGAAAAAAACAAATCA<br>ATCCAAAACGGTATTGCTGTGGAAAGTG<br>TCAAGGCAAGCTGAAATTTATCAGACAG<br>CAGCAGTTTTAAACATTGATAATAAGAA<br>ATCCGCCTAAGGACGGATGGCATGATGG<br>TTAAAATATGCTACACTAATTCTAGTAT<br>CAATGAAAGAGGCTGA
	spv_0803	sans	AACTGCAAAAACGCTTGTTTATCAATGC<br>TTGCATTGTCGTGCACAATATCATAGAA<br>AAAGACGAATCAATACTGAGCGGTATTG<br>CTGCGGACGCTGCCAAGGGAAACTCATT<br>TTTGTCAGACAGTTGCAGTCTTAATGAC<br>TATTTTCGCGAAACAATCAGAAATCCGC<br>CTAAGGACGGATGGCATGATGACTGAAA<br>TGTGCTACACTAGATTCAGTATCAATGA<br>AAGAGGCTTACTGATT
	spv_0803	siz	ACCTCTTAAAAGCAGTAGACGGTCTAAG<br>ATATGCTCCTTGTTCAACGCGTCATCAG<br>ACTTATTGGCTTTATGAATGTTTAAGGT<br>GTCATCACCTTTTTAAACGCAAACGAAA<br>AATAAATACCAATAAGTATTTTTGTGGT<br>AACTGCCATGGTAAGATAAGCTTAAAAA<br>ATCAGTCAAAAGTCTGATTTTTTTTGCC<br>CGTCCATCATTTATAATACAAATAGATA<br>GAATAGGAGGGATAGG
	spv_0803	smb	AAGCAGTGGATGGATTACGCTTTGTGCC<br>CTCCTTGACAAATAGCCACTCTAAACCA<br>CTCAAGCTTTATCGTTGTCAATCCTGCC<br>AACAAAGGTATCAGCGCAAGCGTAGGAT<br>TGATACCAAACGCTATCGCTGTGGACTT<br>TGTCGAGGTAAATTGCTACTAGTAAATC<br>AGCCTGAGGACTGATGAAAGCCTAGTCT

			AACCGTGATATACTATGTCTAACCTAGC AGAAAGAGGAAATATC
	spv_0803	sor	TTTGAAAGAAGTGGATGGACTGCGCTTT GTCCCACCTCTGAAAGACCAAAGTACCT ACCTAGTTTATCAGTGTCAATCCTGTCA GCAAACTTATCAGCGCAAGCGTAGGATT GATACCAAACGCTATCGTTGTGGCGTCT GCCGTGGTAAACTCGTCATCTTAAATCG GCCTAAGGACTGATGTTTGTGAACCCGT TTCATGCTATACTACTTGTAAGAATACC GAAAGAGGAAACAATC
PnpR	*pstS1*	spd	GCACTGGTCTTGGCCTTGCCATTGTCAA AGAACTCAGTCAATTATTAGGTGGCCAA GTCACGGTGACAAGTCAGCTTGGCAGAG GCAGTTGCTTCACGATTTTTCTTCCTAA CCAATCTTTCGCACAGGACTAATAATTT TTAAAGCTATTTTTAGGAGCAGCCATTT TTCAAAAGTGGCTTTTTTTGATTCTTTA CACAATCTTTACATGAATCAGGGATAAA CCTTTACAAAGACTTGTTAGACTATAAA TGTAGTAAGCCTACACAAGAAAAATACA TAGAGATAAAGGTGATTATT
	pstS1	snu	GCACTGGTCTTGGCCTTGCCATTGTCAA AGAACTCAGTCAATTATTAGGTGGCCAA GTCACGGTGACGAGTCAGCTTGGCAGAG GCAGTTGCTTCACGATTTTTCTTCCTAA CCAATCTTTCGCACAGGACTAATAATTT TTAAAGCTATTTTTAGGAGCAGCCATTT TTCAAAAGTGGCTTTTTTTGATTCTTTA CACAATCTTTACATGAATCAGGGATAAA CCTTTACAAAGACTTGTTAGACTATAAA TGTAGTAAGCCTACACAAGAAAAATACA TAGAGATAAAGGTGATTATT
	pstS1	sdg	GTGGAACAGGTCTTGGTCTAGCCATTGT CAAAGAACTCAGTCAACTATTGGGAGGG CAGGTTACAGTCACCAGTCAGCTCGGCA AGGGCAGTCGTTTTACCCTTTTATTGCC AGGACAAATCTCACTTGATTAGTGATGT TTATGGAAAGCTATGTTAGCGCAGTTAA TTGCCTAAGGTAGCTTTTTTTGATTCTT TACATAATCTTTACATGAATCACGGATA AACCTTTACAAAGGCTTGATACACTATA AGTGTAGTAATCCTACACAAAAAATAGT TAGAAATAAAGGTGATTGTT
	pstS1	sds	GGCAAAATGGTGGAACAGGTCTTGGCCT AGCCATTGTCAAAGAACTCAGTCAACTA TTGGGAGGGCAGGTTACAGTCACCAGTC AGCTCGGCAAGGGCAGTCGTTTTACCCT TTTATTGCCAGAACAAATCTCACTTGAT TAGTGAAGTTTATGGAAAGCTATGTTAG CGCAGTTAATTGCCTAAGGTAGCTTTTT TTGATTCTTTACATAATCTTTACATGAA TCACGGATAAACCTTTACAAAGGCTTGA

			TACACTATAAGTGTAGTAATCCTACACA<br>AAAAATAGTTAGAAATAAAG
	pstS1	sig	AGCCGTCAGAGTGGCGGAACAGGTCTAG<br>GTTTATCTATTGTCAAAGAGCTTAGTCA<br>GCTTTTAGGAGGACAAGTCAGTGTTAAA<br>AGCCAGCTTGGTAGAGGCAGTCAGTTTA<br>CCTTAGAATTTCCAAAAGAACTTATTGA<br>AATATAATAAAAGCAGTTATTCTCTAGT<br>TATGAGAATAGCTGTTTTTTTAATCTTT<br>ACAAAATCTTTACAGGAATCCACAAAGA<br>ACTTTTACAAAAGCTTGGTATCCTATAG<br>GTGTAGCAAAACTACATCAAAAAAATCA<br>ATATGATAAAGGTGATCATT
	pstS1	siz	AGCCGTCAGAGTGGCGGAACAGGTCTAG<br>GTTTATCTATTGTCAAAGAGCTTAGTCA<br>GCTTTTAGGAGGACAAGTCAGTGTTAAA<br>AGCCAGCTTGGTAGAGGCAGTCAGTTTA<br>CCTTAGAATTTCCAAAAGAACTTATTGA<br>AATATAATAAAAGCAGTTATTCTCTAGT<br>TATGAGAATAGCTGTTTTTTTAATCTTT<br>ACAAAATCTTTACAGGAATCCACAAAGA<br>ACTTTTACAAAAGCTTGGTATCCTATAG<br>GTGTAGCAAAACTACATCAAAAAAATCA<br>ATATGATAAAGGTGATCATT
	pstS1	std	GCACTGGTCTTGGCCTTGCCATTGTCAA<br>AGAACTCAGTCAATTATTAGGTGGTCAA<br>GTCACGGTGACGAGTCAGCTTGGCAGAG<br>GCAGTTGCTTCACGATTTTTCTTCCTAA<br>CCAATCTTTCGCACAGGACTAATAATTT<br>TTAAAACTATTTTTAGGAGCAGCCATTT<br>TTCAAAAGTGGCTTTTTTTGATTCTTTA<br>CACAATCTTTACATGAATCAGGGATAAA<br>CCTTTACAAAGACTTGTTAGACTATAAA<br>TGTAGTAAGCCTACACAAGAAAAATACA<br>TAGAGATAAAGGTGATTATT
	pstS1	splr	GAGGAAAAATTAGTGTTAACAGTCAAGT<br>GGGACAAGGATCAACCTTTACCCTAAGC<br>TTACCAATTACGATTGGTATGGGAAGAT<br>GACGTGTCATATCAGAAAAAGATCACCG<br>ATCGTGGTCTTTTTTTTGATGGCGATAT<br>TATTGTAAAAGGAAATGTTCAATCTTTA<br>CACAATCTTTACAATAATCCCTAATATT<br>TCTTTACATTTGCCTTATATACTAAAAA<br>TGTAAACAAGAGGAACATCAAAAAATAA<br>CAGGACTGTCTCTTCAAATGATTGTTAA<br>AAGAGAAAGTGAGTTTACGG
	pstS1	seu	GGGACGTAGCAGGCAAAGTGGTGGAACA<br>GGCCTAGGCCTAGCCATTGTAAAGGAGC<br>TGAGTCAGCTTCTTGGCGGTCAGATAAC<br>AGTCACAAGTCAGCTAGGAGAGGGCAGC<br>CAATTTACCTTATTCTTACCAGAAGCTA<br>TCAACAAGTAAACAGTCACTACCAGCTA<br>CTTAAGGCCAGCCTTGGTAGTGGCTGTT

			TTAGGTTTTACAAAATCTTTACATTAAT CAGGGATAAACCTTTACAAAGACCTGAT AAGCTATAGGTGTAGCAAGCCTACAACA CAATATCATAAAAGATAAGG
	pstS1	sub	GAACAGGATTAGGTTTATCGATTGTTAA AGAACTCAGTCAGGTTTTAGGAGGCCAG GTTTTCGTAAAAAGTCAGATTGGAAAAG GTAGTCATTTTACCTTAGAATTTCCTAA GTCCATAACGTCATAAATGTTAATATTT TAGTACCATTTAGATTTTAAAATGGTAC TTTTTTTATACAAATATAAATCTTTACA AAAACTTTACAATAATCACTTATAAATC TTTACAAAGACTTGATAGACTATAACTG TAGCAAACCTACACAAGAAATTTATCAA ATCAAAAAAAGGTGAAAATT
	pstS1	spoc	AGTGGAGGGACTGGCCTAGGTCTTTCGA TTGTAAAAGAACTGACTCAGGTCTTAGG GGGAACAGTTACTGTAAGAAGTAAGCTT GGAGAGGGTAGCCAGTTTACCATAAGCC TGCCCTACTATCTAATAAAGGAAGTCAA GTGATTAAAAAGAGTTTATTATAACTCT TTTTTTATGCTCTTAGCATAAGATTTAC AAAATCTTTACATGAATCACTACTAAAT CTTTACAAATACTTGTTAGACTATAAGT GTAGTAAAGCTACAGAAAAACTTTATTA AATATAAAAAGGTGATGATT
	pstS1	surn	GAACGCTTTTATAGAGTTAATAAAGGCA GAAGTAGGCAGTCTGGTGGTACAGGACT TGGTTTATCAATAGTTAAAGAATTAACA CAAGCATTAGGTGGTAAAATAAAAGTAT CAAGCCAATTAGGTGTTGGAACACGATT CACACTACAATTACCATTGCAGTTTAAA GAAAAAAATGAAGCAGTTACTATGTAAA TGCTTCATTTTTTATTTCTTTACACAAT CTTTACAAAAATCCATAAATAACCTTTA CAAAGACTTGATAAACTATAGTTACAAA AAATAAAGAGGTTATCGAAA
RR07	*endoD*	spd	TTTTCCTCCGAAAAATAATCGCGGAATA TCTCCTATTCTATTTTAAAGGATAAAAG TAAATCTAACAAGTGTTAATGTCAAGTT TGTAAAAGAAAATTATAATTGATAAGTG TATTATTTTTAAGAGTTTTTAACCTTAT TTTAGATTTGTGCAAACAGTTATTGTTT TTTGTCTAAAAATGTTTTAGGATAAAAA TGTGAT
	endoD	std	TTAAGCTAAGATTTTAGAAAGAAAATTC GTGAGAAAAGGCCTTAATTATATTAATT TTACCAATTTCTAACCTAGATATAGATA TTTCCTTTTATATATTGACATTTAAAAG AAATATTTTAAAATTAGGGAGAAATTAT CTTAATCCCTGTGATTGGGATAAAAATT TTACGAAGAAAGTTAAGCGCTTTCTTTA

			AAATAATAAAATCAATCTTTTAGGAGGA GAAAATGAAGAATTCA
	endoD	smb	TTTTTCCTCCGAAAAATAATAACGGAAT TTCTCCTATTCTATTTTAAAGGATAAAA GTAAATCTAACAAGTGTTAAGCTAAGAT TTTAGAAAGAAAATTCGGGAGAAAAGAC CTTAATTATATTAATTTTACCAATTTCT AACCTAGATATAGGTATTTCCTTTTATA TATTGACATTTAAAAGAAATATTTTAAA ATGAGGGAGAAATTATCTTGATCCCTGT GATTGAGATAAAAATTTTACAAAGAAAG TTAAGCGCTTTCTTTAAAATAACAAAAT CAATCTTTTAGGAGGAGAAA
	endoD	sor	CTCCTATTCTATTTTAAAGGATAAAAGT AAATCTCACAAGTGTTTAGCTGAAATTT TCATAAGAAAGAAGAACTTTCTTTTAAA ATGAGATCTAGTTTCCCTTTAAGATAGA TGATTTATAGGATTGTGTCCAAATTTTA CTGATTATTATCCTAGTTATAGTTTTTA TGTTAAATAGATTGACATCGCTTTCATA TAGGATAAAATGAAGATGATCCTGTTCT GGTGGTGAGTACTGAGAACAGTTACTTT TTATCAAAAAAGTAAAGCGCTTACTTTA TATTTTATTAGGAGGATAAG
	endoD	ski	TATGACAACGAAATGTCTTACACTGCAC AACTTGTACGTACTCTTGAATACTTCGC AAAAATTGCTAAATAATTCATGTGTATG ATGAGAGGAGGGTGACCTCCTCTTTTTT TGTACTTTTTTTAGTTCCTCCTCACCTT ATTATAGGATTTGCTAAAATGGATTGAC AGACCTCTCTTTAAAGGGGTAAACTAAA TTTGAGTGAAGATTTCCAATTAAACAAA ATGTAAGCGATTGCACGAGAGCAAAGGA GAAGTGTTTTAGGAGATTGGAAATTAGT AAAATAAGATGGAGGTCAAG
	endoD	sip	TTGTTTCATGGTATGACAACGAAATGTC TTACACTGCACAACTTGTACGTACTCTT GAATACTTCGCAAAAATTGCTAAATAAT TCATGTGTATGATGAGAGGAGGGTGACC TCCTCTTTTTTTGTACTTTTTTTAGTTC CTCCTCACCTTATTATAGGATTTGCTAA AATGGATTGACAGACCTCTCTTTAAAGG GGTAAACTAAATCTGAGTGAAGATTTCC AATTAAACAAAAATGTAAGCGATTGCAC ATGAGCGAAGGAGAAGTGTTTTAGGAGA TTGGAAATTAGTAAAATAAG
	endoD	saup	ATGACAACGAAATGTCTTACACTGCACA ACTTGTTCGTACTCTTGAATACTTCGCA AAAATTGCTAAATAATTCATCTGTATGA TGAGAGGAGGGTGACCTCCTCTTTTTTT GTACTTTTTTTAGTTCCTCCTCACCTTA TTATAGGATTTGCTAAAATGAATTGACA GACTCCTCTTTAAAGCGGTAAACTAAAT

			CTGAGTGAAGATTTCCAATCAAACAAAA ATGTAAGCGATTCCACATAAGCAAAGAA GGAGTGTTTTAGGAGATTGGAAATTAGT AAAATAAGATGGAGGTCAAG
	endoD	sgo	TTACACTGCACAACTTGTACGTACTCTT GAATACTTTGCGAAAATCGCTAAATAAT TCATAGTATAGAAGAAGAGAGCTTGGCT CTCTTTTTTTGTACTTTTTTTAGAGTTT TAAGCTCTATTTATAGGATAAGGTGGAA GGGATTGACAATGAAAGTGAGCAAAGGG TAGAATGGTTTTGAGTGAAGTTTTCTTT TTTTAAAGAAAAATGTAAGCGATTACTC AAAAAGGGAGTGGTGTTAGTATTTAGAA TTGGATAGTCGCAAAACTAAGAAAAGAA GAGAAGGAGTCAAGGTAAAC
	endoD	ssus	GGAACGCCTGGATGAACTGGTAGGTTGG TGTATTAAGTTTGTAGAAAATAATTAAC GAAGACAACGAGGAGGGAAACTTCCTCT TTTTTGTTTTCTCTAAACTAAAGTTCTT TTTATCTGATTTAAAGAAGGGAACCATG AGGGCTTGAAGGTCATATTTTTCCTCTG TACAATGGAATTAATAAAACCTGATAAA CTTCGTGTGATAGCTGTTCTATCAAAGG TAGCATGTGCCAACTATGATCTGGAAAG GAGGAAATTGAGAACAGCTGTTGCACTA CAATTGTGTAGGAGGAAAAT
	endoD	sib	ATATTTGCCCTCTATTTTAAGAAGTGAA AGTTTTAGCATTGATTAAAGCGCTTTCT CGGCATAAAATGGACATGGGAAGTCGCA CATGTCTATTCCATTTCTGGAACGAAAG TGCATATTATGGATTATCTTACTATTGT CAGTCAATCGTCCAATCATTGGCTAAAT AAATTGATAATAGAAAATAGTGAACTCA GTATCATACATCATCTAGTAATTGATAG AGTAGGAGGTAAATGTAAAAGCACTGGG AAAAGTGTGATGTGAGACTACTATTTCA AATTAAGAAAGGAGAAGATG
	endoD	scf	CGGATCATTGTTTGACGCAACTCAAACT AAAGTTCTTGACGTTGACGGTAAACAAT TGGTTAAAGTTGTTTCATGGTATGACAA CGAAATGTCTTACACTGCACAACTTGTT CGTACTCTTGAATACTTCGCAAAAATTG CTAAATAATTCATGCGTATGATGAGAGG AGGGAAACCTCCTCTTTTTTTGTACTTT TTTTAGGGCTTTCTCATCATATTATAGG AATTGCCAAAATAGATTGACAGTGCTAA ATAGATTAAGGTATCCTATTCTTGATTG GAGATTTCTAATACAACGAA
	endoD	svf	ATGACAACGAAATGTCTTACACTGCACA ACTTGTTCGTACTCTTGAATACTTCGCA AAAATTGCTAAATAATTCATCTGTATGA TGAGAGGAGGGTGACCTCCTCTTTTTTT GTACTTTTTTTAGTTCCTCCTCACCTTA

			TTATAGGATTTGCTAAAATGAATTGACA GACTCCTCTTTAAAGCGGTAAACTAAAT CTGAGTGAAGATTTCCAATCAAACAAAA ATGTAAGCGATTCCACATAAGCAAAGAA GGAGTGTTTTAGGAGATTGGAAATTAGT AAAATAAGATGGAGGTCAAG
RR08	*pavB*	spd	GAACAGCGGAGCATCTGGCAAAAAAACG CCAATTGTGGACCTATATTCAGCAGAAA AATCCAGAAGTCTTTCAGGCTATTCGTA AGACCATGTTGAGCCGTTTGACCAAACA TTCTGTCTTGCCAGATCGCAAACTGTCC AATGTCGTCTATCAAATCACCAAATCTG TTTATGGATTTAATTAATATAAGTGTTT TATAAGAGGGATTTAAGAAAAATTTTAA CTTTTTCTTAGTCCTTTTTAATTTCAGG AGATTATACTAGAGTCATCAAATAAAGA AAGACTCTAAGGAGAATCCT
	pavB	smb	GAACAGCGGAGCATCTGGCAAAAAAACG GGAGCTGTGGACCTATATTCAGCAGGAA AATCCAGAGGTTTTTCAGGCTATCCGCA AGACCATGTTGAGTCGTTTGACCAAACA TTCAGTCTTGCCAGCTCGCAAACTGTCC AATGTTGTCTATCAAATCACCAAATCTG TTTATGGATTTAATTGATATAAGTATTT TATAAGAGGGATTTAAGAAAAATTTTAA CTTTTTCTTAGTCCTTTTTAATTTTAGG AGATTATACTAGAGTCATCAAATAAAGA AAAACTCTAAGGAGAATCCT
	pavB	siu	AATAGGTTTGCTTTTTAACATAATGAGT TATGGATGGGAAGTCTAGGATTTTGTTG CTAAAGAGTGGTTCTATTTGTTAGCAGT ATAAATGGAATGAGCAGGGAAGAGTAAG TAATATGTGTTTTTTGTGAATAATATTG TAGTAAAAAAGAAATATTGCCGATATTT CACTATTTTTCTTTAACATAATGAAATG ATAATTTCTATTTAAGAAAAATTTTAAC TTCTCCTTACAGATAGTTAATAGGAGCT TGCTATACTTTAGTCAACCATTAAAAAT AAATCAAAGGAGTAAATAAT
	pavB	sor	TCAACGTACTTGGCTTTCGTTCCTACGA AAATGCTCCTCTGGATGTCCAATTCGGA CCCAATGACCTCTTGCAGGCAGATGCCT ACATCATGGCCGTCATTAATAACCAACA TTTGGACACCTTGGCAGAGCTAAGTGAG TAGGAGAGGAAGGGCACTCTCTTTCCAA ATAATTAACGAGTCAATATAAGTATTTT ATAAGAGGGATTTAAGAAAAATTTTAAC TTTTTCTTAATCCTTTTTAATTTTAGGA GATTATACTAGAGTCATCAAAAAAAAGA AAAACTCTAAGGAGAATCCT
	pavB	spei	GGACTAGAATAAATAGCAGAGATTGGCT TATTCTTTAGTATTTTTAATTGTGAGAG GGAAGTGAGTCCCTTTTGTGACAAAGAG CGTGTCCATTCGTCAGGTGTGTAAGTTG

			AGTGCGCGTGACGGACAAAATAATAAAT<br>GTTGTTCATGTAATCTAGTATATCCCAA<br>CATGTGAAAATTCTATAAATAAGCAAGA<br>AATAATGCTTATTTAAGAAAAACTTTAA<br>CTTTGTCTTACGGATAGTTAATAAGAGC<br>TTGATAAACTTTAATCAACCATTAAAAA<br>TAATCAAAGGAGTAAATAAT
	pavB	siz	TGGGAGCAATCTTGGTAACCATGAACCC<br>ACTTGATTTCTTCAAAAAGCCAGAAACC<br>ATTGAAGAACAATTAAGCCTTGATAATG<br>AGTAAGAAGTCTTAGTTATTGGGTATGA<br>TGTAAAATGACAAAAAGCCTTTCTCAAA<br>GGCTTTTTGTTAGGTGTGAACTAGCCTC<br>TTCTCATTTTTTGTTTCTTTTAAGACTT<br>AATAAATCCATAAGCACTAATTAAGATT<br>TAGTTAATAAATAATGTAGAATGCCTTA<br>AGAAGTGCTTGTTATACTAAGGTCACTT<br>AAAAGAAAAGAGGACATTTT
	pavB	sgw	GAACAGCGGAGCATCTGGCAAAAAAACG<br>TGAGCTATGGACCTATATTCAGCAGGAA<br>AATCCAGAGGTCTTTCAGGCTATCCGTA<br>AGACCATGTTGAGTCGTTTGACCAAACA<br>TTCAGTCTTGCCAGCCCGCAAACTTTCC<br>GATGTCGTCTATCAAATCACCAAATCCG<br>TTTATGGATTTAATTAATATAAGTATTT<br>TATAAGAGGGATTTAAGAAAAATTTTAA<br>CTTTTTCTTAATCCTTTTTAATTTTAGG<br>AGATTATACTAGAGTCATCAAATAAAGA<br>AAAACTCTAAGGAGAATCCT
	pavB	ssa	TGCCAGCTCTAGTTGGAATCCTACTTTC<br>TCTCTTTTTGCCAAACAAGCAGAAGAGT<br>GAGCGTTTTGAGATGGAGAGCTAATAGA<br>TAACATCCGCCACAATGTGGTGGATTTT<br>TTTGACTCATTGTCAATTATAGTAGGGA<br>ATTTATGGCGAAGGAATTCAAGCTGTTT<br>TTTTATGAGCAATATGACAATATAATCC<br>TTTTATAAGATAGATTTAAGAAAAATTT<br>TAACTTTTTCTTAACCATTTTTAATCTT<br>AGGAGATTATACTAAAGTCATAAAAAAA<br>CAAACCTAAAGGAGAATCTT
	pavB	sag	CGTTGATTTTGTCGATGTTGACTTATTG<br>GATATTTCTAGAGAAGCACACCAACAAA<br>CAGGTTTTGAAGTGACTAGAGTTCAGTT<br>AGTAGCTTATGGTATCTGTCCGGAGTGT<br>CAGAGGAAGCGTAAGGATAAATATAAAA<br>AAGATTGAAACGTAATTGTTCAATCTTT<br>TTTTGTTTATAGACTAAGAGAAAAAGTT<br>TAAGTTTTACTAAGTAATAATTAAGAAA<br>TAATTTATAATTGCTTAAAAAATGATTT<br>ATTTTTTGTTGGTATACTCTCATTAACA<br>AAAAGAAAGAGGAATCCCTT

	pavB	lli	CGCACCACGTAAATCAGCTAAACCTTCA<br>TACGATTTGCCTGAAACACAAGAAGGCT<br>TCTCACTTGCTGATTTCCTTGGTGAAGA<br>CTTCGATATTAACGATTTGTAAGAAAAA<br>TTCAAAAATATCCTCTAAAAGACCCGTT<br>GGGTCTTTTTTTGTATAGAAAACTAAGA<br>AACAATTAAATTGCAAAGTTTGATAATT<br>TATAAATTTTATGGGAAATTCACAGAAA<br>TCTAACATAAAAACTAGTAGTTCTCCTG<br>TTAAATTTTTTTATAATAAGGATGTAAA<br>AAATAAAGGAGAACTACAAT
	pavB	gha	AATAATTAAGAAATATTGCTCTTCACTT<br>CTTCGCTTTTTAAAACTGTTAAAGAAAT<br>ATTAACATAAGATATTTTCTTTGTCAAC<br>ACTTTTTAGTAAAAAAATTAAAAACTTT<br>TTTCGAAATTGTCAGAAATCTAATTTAA<br>ACCATATATATTAGTATATTTTTAAAAT<br>AATTATTTTATAATATCTATTTAAGAAA<br>TATTTAAACATTTACTAATAGTTAGTTA<br>ATATCTATAAGGTATACTGTATTCATCA<br>AACAAAAAGTTTTGGTAATAAAGTATTA<br>ATAAGAGGAGAAAAATACAA
YesN	*ccdA-1*	spd	CTTTTGTGCTTTGACAGTTTTTTGTATA<br>GTTTTTGTCCAAGATTTCTAAATCATCT<br>GATTAAATTCCTATTTTCCCCTATCATT<br>GTCCCTGTTTTTTCGAAAATGGAAGGAG<br>TATAATAA
	ccdA-1	ssa	TACCATAAATCCACAATAAGTGATAAAC<br>ATTTTTAACTCTTGATTTAGCAAAGATA<br>AAACCAAATTCAAGTTTTTTCAAATCTT<br>TCTCTCTATTTTTAGGGGGATTTTTATT<br>TTATACTA
	ccdA-1	seq	TCCTGAGCCCTTGTTTGCTGTAGCTGAT<br>AGGATTTTGCTGATGGCCTAGTCAGGCA<br>TATCGTATCAAATTCAAGTTTTTTCATG<br>TCACTGTCTATAGTTTGTCTGCCCTACT<br>TTAGGTAG
	ccdA-1	sans	TGAAATGGAATAATTAAAAATATCTATC<br>ACGAAAAGTGATAGTTTTTTTATGACCA<br>TATCAAATTCAAGTTTTTACAAATCGTA<br>TCCTCTATTTTTAGATAGTGAACCAGTG<br>TATACTTT
	ccdA-1	spei	CACATTTGAAATGGAATGATAAAAAAAC<br>TATCACGGAAGTGGTAGTTTTTTTATGA<br>CCATATCAAATTCAAGTTTTTACAAATC<br>GTATCCTCTATTTTTAGATAGTGAACCA<br>GTGTATAC
	ccdA-1	smb	ATAAGATAGCTCACCTTTTGTCTCCCTT<br>CTTCTTTTTATTTTTTGAAGTATAATCT<br>GATTAAATTCCTATTTTTCCCTATCATT

			GTCCCTGTTTTTTCTTGATTTTTGGAGC<br>TATAATAG
	ccdA-1	sor	TTCTATCAAAGTCAGACCTTGTCTGGCT<br>TTTTTTGTGCAAGATTTCTAAATAAACT<br>GATTAAATTCCTATTTTTTCCTATCATT<br>GTCCCTGTTTTTTCTAGAGTTTTGGAGC<br>TATAATAG
	ccdA-1	sgw	TTTCTATCAAAGTCAGACCTTGTCTGGC<br>TTTTTTTGCACCTTTTTTTAAATAATCT<br>GATTAAATTCCTATTTTTCCCTATCATT<br>GTCCCTATTTTTTCTTGATTTTTGGAGC<br>TATAATAG
	ccdA-1	scai	GATAGCATCAAACAACTTTAGGGAGTTT<br>CTAAGGTTGTTTTTTTCTTTTTATAAAA<br>TAGCGTATCAAATTCAAGTTTTTGCATG<br>TGTCAGTCTATGGTTTGCTGTCTTGGTT<br>TCAAGTAA
	ccdA-1	spf	ATCTCATTAAAAGAGAGGTCGGGTTTTT<br>ACATGCCTTTAGTCAACCATTTATCTGA<br>TAGCGTATCAAATTCAAGTTTTTGCATG<br>TTCTAGTCTATGGTTTGCGTCACTAGCT<br>TTCGGTAA
	ccdA-1	saup	CGATATAGTTTGTACTTGAAAGTATGAG<br>CTGATGTTGGTAGATCGGAATATTCATC<br>TTCCAATCAAATTCAAGTTTTTGCATAT<br>CAATCTCTCTATTTTTAGAGAGATTTTT<br>TCTTTATA
	ccdA-1	soz	GGGTTTTTACATGCCTTTAGTCAACCAT<br>TTATCTGATAGCGTATCAAATTCAAGTT<br>TTTGCATGTTCTAGTCTATGGTTTGCGC<br>CACTAGCTTTCGGTAAACTAAGGGTAAC<br>TTAAGAGA
RR11	*spv_1803*	spd	AGGCCTACTGTTATAGATTCAATACACT<br>ATATATGTGTTTGTCTGATAAAAATTTC<br>TACTCTTTTTGATTTTAAATAAGTATTA<br>GTTTACATTATGGTGTGAATTGGGTTTG<br>ATATCTCTTTTGAGGAAGTTGCCTTAGA<br>TTTTTCTGATTGTGTTTTATTGTATAGT<br>GTATCTTGCTTGTTTTGAACAGAATTTT<br>TATGACATTTGTCATATTTTCTAGTGAC<br>AGAAGCTTCTGCCTCCTCTGATTTTAAA<br>AGACTATAATTGTAGTATGAAATGGGGG<br>AAGAAGAGATGAGAAATAAA
	spv_1803	scf	AAATTGCTCTCAACCAGTGTGATTGACC<br>TCAACCAAATTGATGCCAAGTCTCTAGA<br>AGAAATTTCTTACTTCAAAGATGCTGGT<br>CTCAATGATTTGAAAAACATGAAGGCGG<br>ATGCCTTGGTGAAGGCCTTGAAATTGCA<br>TGGCATGAAAGAAGAAGGCCAGTAAGAA

			GAATAGAAAAAGTCGAGTGGAGTCACTT AGCTTTTTTTAGTGACTTTTGTCATGTG AGCGAGTGACAAAATCATCTAGTTGCCT CTTCTTCCTTTTCATATAATAAAGTCAG AAAGTAAGAGGTAAGAAAAA
	spv_1803	svf	AAATTGCTCTCAACCAGTGTGATTGACC TCAACCAAATTGATGCCAAGTCTCTAGA AGAAATTTCTTACTTCAAAGATGCTGGT CTCAATGATTTGAAAAACATGAAGGCGG ATGCCTTGGTGAAGGCCTTGAAATTGCA TGGCATGAAAGAAGAAGGCCAGTAAGAA GAATAGAAAAAGTCGAGTGGAGTCACTT AGCTTTTTTTAGTGACTTTTGTCATGTG AGCGAGTGACAAAATCATCTAGTTGCCT CTTCTTCCTTTTCATATAATAAAGTCAG AAAGTAAGAGGTAAGAAAAA
	spv_1803	sig	AAAAAATTAGCCTCAACGGTCTCTGTTG ATATGGAAACTATTGATTGGGACAAGGT TAAAGACCTATCTTACTTTAAAGATCTT GGTATCGGTGACCTAAAAGACATCAAGG CATCGACTGTTTTCACAGGATTAAAACT CCACGGTTTTAAAGAAGAAACGCCAGCA CCATAGGTTCGGACTCTTGTCCGGCCTT TTTTATTTTCCATGACATTTGTCATGTC AGCCAGTGACAAAATCATCTAGTAGCCT GTTATTCCTTTCGCTATAATAAAGTCAG AAAGTAAGAGGTAAGGAAAA
	spv_1803	saup	AAAAAATTAGCCTCAACGGTCTCTGTTG ATATGGAAACCATTGATTGGGACAAGGT TAAAGACCTATCTTACTTTAAAGATCTT GGTATCGGTGACCTAAAAGACATCAAGG CATCGACTGTTTTCACAGGATTAAAACT CCACGGTTTTAAAGAAGAAACGCCAGCA CCATAGGTTCGGACTCTTGTCCGGCCTT TTTTATTTTCCATGACATTTGTCATGTC AGCCAGTGACAAAATCATCTAGTAGCCT GTTATTCCTTTCGCTATAATGAAGTCAG AAAGTAAGAGGTAAGGAAAA
	spv_1803	slat	AAAGAAGAAGGTCAGTAAGAAGACAAAA AGAGAGTGGGACAGAAATCGGTCATTCG TTAGAATTCGATTTCGTCGTCCCACCTC CGCACAGTTGAGTAGGGCTGTAAAAGCT GATGAAATCAGCGTAGTAGAGCCCACTC AACCACTGCGTCTTGCTCGACAATCCAA AAACAAGAAGAGGCTAGGACTTTTGTCC CAGCCTTTTTTTAGTGACATTTGTCATG TGAGCCAGTGACAAAATCATCTAGTGGC ATTTTTTGTCTTTCGCTATAATAAAGTC AGAAAGTGAGAGGTATAAAA
	spv_1803	stra	AAAAATTAGCCTCAACGGTCTCTGTTGA TATGGAAACTATTGATTGGGACAAGGTT AAAGACCTATCTTACTTTAAAGATCTTG GTATCGGTGACCTAAAAGACATCAAGGC

			ATCGACTGTTTTCACAGGATTAAAACTC<br>CACGGTTTCAAAGAAGAAACCCCAGCAC<br>CATAGGTTCGGACTCTTGTCCGGCCTTT<br>TTTTATTCTCCATGACATTTGTCATGTC<br>AGCCAGTGACAAAATCATCTAGTGGCCT<br>GTTATTCCTTTCGCTATAATATAGTCAG<br>AAAGTAAGAGGTAAGGAAAA
	spv_1803	smb	TTAAGAAAGTAATTGAAGAGTTTTTTAC<br>TCAGCCTATAATATATGTGCTTATGGGA<br>TGAACATTTCTGATCTTTTGATTTTGAA<br>AGTGTATTAGTTTACGTTATAGTATGAA<br>TTGGATTTAATATCACCTTGAAGGAAAT<br>AATTATAGATTCTTTTCTATCCGTGTTT<br>TGCTGTGTAATGTTTCTTGATTCTTTCA<br>AATAGAATCTTTTATGACATTTGTCACA<br>TCTCCTCATGACAGAAGATTCTAGTCGC<br>AGTAGATTCAAAGAATTATAATAGATGT<br>ATCAAATGGAGGAAGAAAAA
	spv_1803	sgw	ATGAAGATAGTGAAATCGTCACCTTCTA<br>TGTTGGTGAAGACGGAAGCGAAGAACTT<br>GCCAATGAAATTGCCCAAGAAATCGCAG<br>AAGAATTCGAAGATGTTGAAGTAGAGAT<br>TCACCAAGGCCAACAACCAGTTTACCCA<br>TACCTATTTAGTGTGGAATAAAAATTTA<br>ATAGATTAAAAAGAAAGTTGATTTTACA<br>ACTTTCTTTTTTTATGACATTTGTCATA<br>TTTCCTCATGACAGAAGCTTCTAGTGAC<br>TATACCTTCAAAGAATTATACTAGATGT<br>ATCAAATGGAGGAAGAAAAA
	spv_1803	stv	AAAAGAACTTTCAGTACCTGATTCGAAA<br>CCAGATCAATTGACTCAAAACATCTCTG<br>TAGAAAAAAATCAACTTCCAAATACAGG<br>AAGTCAAGAAGATGGTTTGAAAAATCTA<br>GGAATTCTAACAACCCTAGCAGGTGTCA<br>TGACACTTGGATTGCTAGGTAAAAAGAA<br>ACGAAACGATGAATCAGACTAATCATTT<br>TTAAGAACCGAGAAATCGGTTCTTTTTT<br>ATGACATTTGTCATATTTCCTAGTGACA<br>GAAGCATCTAGCTCCGCCAAATTCAAAA<br>GACTATAATTGAAGTATGAA
	spv_1803	sor	GAAACATATGTTGGACGAAGACAGTGAA<br>ATCGTGACTTTCTATGTCGGTGAAGACG<br>GAAGCGAAGAACTTGCCAATGAAATTGC<br>CCAAGAAATCGCAGAAGAATTTGAAGAT<br>GTTGAAGTAGAGATTCACCAAGGTCAAC<br>AACCCGTATATCCATATCTTTTCAGTGT<br>GGAATAAAAATTTAATAGATAAAAAAGA<br>AAGTTGATTTTACAGCTTTCTTTTTTTT<br>ATGACATTTGTCATATTTCCTAGTGACA<br>GAAGCATCTAGCTCCGCCAAATTCAAAA<br>GACTATAATTGAAGTATGAA
	spv_1803	std	GAAATTTAACAGATTTTATAACTCTTAT<br>AAATTTTAGGGTTTTGTTCAACGTATAA

			TATATATGTTTGCCTGATAAAAATTTCT<br>ACTTTTTTGATTTTAAATAAGTATTAGT<br>TTACATTATGGTGTGAATTGGGTTTGAT<br>ATCTCTTTTGAGGAAGTTGCCTTAGATT<br>TTTCTAATTGTGTTTTATTGTATAGTGT<br>ATCTTGCTTGTTTTGAACATAATTTTTT<br>ATGACATTTGTCATATTTTCTAGTGACA<br>GAAGCTTCTATCTCCTCTGATTTCAAAA<br>GACTATAATTGTAGTACCAA
	spv_1803	strn	GCTGATTCGAAACCAGATCAATTGACTC<br>AAAACATCTCTGTAGAAAAAAATCAACT<br>TCCAAATACAGGAAGTCAAGAAGATGGT<br>TTGAAAAATCTAGGAATTCTGACAGCCC<br>TAGCAGGTGTCATGACACTTGGATTGCT<br>AGGTAAAAAGAAACGAAACGATGAATCA<br>GACTAATCATTTTTTAAGAACCGAGAAAT<br>CGGTTCTTTTTTTATGACATTTGTCATA<br>TTTCCTAGTGACAGAAGCATCTAGCTCC<br>GCCAACTTCAAAAGACTATAATTGAAGT<br>ATGAAATGGAGGAAGAAGAA

Table S8. Input and output results from motif scanning using FIMO (Supplementary file). List of all binding sequences discovered in upstream regions of RR-specific differentially expressed operons from RR overexpression mutants, using the GLAM2-predicted motifs. Additionally, the Excel file includes input motifs and sequences.
